# The impact of semaglutide on liver outcomes in patients with or at risk of MASH: a dose and duration response meta-analysis of randomized trials

**DOI:** 10.1186/s13098-025-01995-z

**Published:** 2025-11-24

**Authors:** Ranran Kan, Siyi Wang, Xiaoyu Meng, Yaming Guo, Danpei Li, Xuefeng Yu

**Affiliations:** 1 Division of Endocrinology, Department of Internal Medicine, Tongji Hospital, Tongji Medical College, Huazhong University of Science and Technology, Wuhan, China; 2 Branch of National Clinical Research Center for Metabolic Diseases, Wuhan, Hubei China

**Keywords:** Semaglutide, Metabolic dysfunction-associated steatohepatitis, Systematic review, Meta-analysis

## Abstract

**Background:**

Metabolic dysfunction-associated steatohepatitis (MASH) is a progressive liver disease associated with significant morbidity and mortality, yet management options remain limited. Although glucagon-like peptide-1 receptor agonists such as semaglutide have shown benefits in metabolic health, their efficacy and safety in patients with MASH require further elucidation.

**Methods:**

We systematically searched PubMed, Embase, the Cochrane Library, and ClinicalTrials.gov for randomized controlled trials (RCTs) evaluating semaglutide in patients with MASH from inception through August 23, 2025. This meta-analysis was conducted in accordance with the Preferred Reporting Items for Systematic Reviews and Meta-Analyses (PRISMA) guidelines.

**Results:**

A total of 22 RCTs involving 32,013 patients were included. Semaglutide significantly improved MASH Resolution (RR = 1.98, 95%CI: 1.57 to 2.50) but did not yield significant improvement in Fibrosis Regression (RR = 1.18, 95%CI: 0.74 to 1.88). Semaglutide also reduced Liver Steatosis (WMD = -11.30%, 95%CI: -18.70 to -3.91) and the Enhanced Liver Fibrosis (ELF) score (WMD = -0.49, 95%CI: -0.70 to -0.29). Significant reductions were observed in liver enzymes, including alanine aminotransferase (ALT; WMD = -5.55 U/L, 95%CI: -9.21 to -1.89) and aspartate aminotransferase (AST; WMD = -3.85 U/L, 95%CI: -7.67 to -0.03). Additionally, semaglutide improved weight management, glycemic and lipid parameters, and reduced all-cause mortality (RR = 0.82, 95%CI: 0.74 to 0.91) and cardiovascular risk (RR = 0.83, 95%CI: 0.75 to 0.92). Subgroup analyses revealed the greatest benefits in patients receiving higher doses (≥ 2.0 mg weekly) and longer intervention durations (≥12 months).

**Conclusion:**

Semaglutide represents a promising pharmacotherapeutic option for MASH, demonstrating significant improvements in histologic resolution, liver injury biomarkers, and metabolic parameters, particularly at higher doses and longer intervention durations, though its effect on fibrosis regression remains limited.

**Supplementary Information:**

The online version contains supplementary material available at 10.1186/s13098-025-01995-z.

## Introduction

Metabolic dysfunction-associated steatohepatitis (MASH), previously known as non-alcoholic steatohepatitis (NASH), represents the progressive inflammatory form of metabolic dysfunction-associated steatotic liver disease (MASLD) [1]. It is characterized by the accumulation of fat, hepatocyte injury, and lobular inflammation. Without intervention, MASH carries a significant risk of progression to fibrosis and cirrhosis [2]. Importantly, growing evidence recognizes MASH as an independent risk factor for hepatocellular carcinoma (HCC), which can develop even in the absence of advanced fibrosis or cirrhosis [3]. As a condition affecting a substantial portion of the global population, MASH has emerged as a leading cause of chronic liver disease worldwide and is strongly associated with obesity and type 2 diabetes (T2DM), with contribute to increased cardiovascular morbidity and mortality [4].

Currently, resmetirom, a thyroid hormone receptor agonist, remains the only pharmacotherapy approved for MASH. Nonetheless, many patients struggle to achieve or maintain necessary lifestyle and weight-loss goals [5]. Although numerous biological targets are under investigation, most potential agents have not shown strong efficacy in late-phase clinical trial [6].

Glucagon-like peptide-1 receptor agonists (GLP-1 RAs) have attracted considerable attention for their potential in the management and prevention of MASH progression. Their mechanisms of action, including promoting weight loss, improving glycemic control, and exerting potential direct anti-inflammatory and anti-fibrotic effects, align closely with key pathogenic drivers of MASH [7]. Liraglutide, a first-generation GLP-1 RA, has demonstrated efficacy in improving liver enzymes and facilitating the histological resolution of MASH [8]. However, evidence for other GLP-1 RAs, particularly regarding their dose-response relationships and histological impacts, remains limited and controversial [9].

Semaglutide, a second-generation GLP-1 RA, is distinguished by its superior efficacy in weight reduction and glycemic control, coupled with its cardiovascular benefits [10, 11]. Evidence from murine models and early human studies suggests potent benefits on liver injury [12, 13]. However, phase 3 trials specifically in MASH populations have been constrained by small sample sizes, varying and heterogeneous endpoints, and short study durations, leaving critical questions unanswered.

Crucially, the hepatoprotective effects of semaglutide are not fully elucidated, particularly the optimal dosing strategy and the intervention duration required for hepatoprotective benefit in MASH. Furthermore, given the pathophysiological overlap between metabolic conditions, patients with obesity and T2DM represent a large at-risk population for MASH [14]. Semaglutide is widely used in these groups, and liver-related outcomes are frequently reported as secondary endpoints in their RCTs [14].

This context provides a unique opportunity to integrate evidence across metabolic disorders through a pooled meta-analysis, enabling a robust and hypothesis-generating examination of semaglutide’s effects on hepatic and metabolic parameters. Therefore, we conducted this comprehensive systematic review and meta-analysis to synthesize available evidence from RCTs to clarify the impact of semaglutide on liver outcomes, with particular emphasis on dose-response and intervention duration relationships. Our aim is to provide timely insights into the potential clinical application of semaglutide for MASH.

## Methods

This research was conducted in compliance with the PRISMA guidelines [15]. The protocol for the review was registered with PROSPERO (number CRD 42023471198).

### Search strategy

We systematically searched PubMed, Embase, the Cochrane Library, and ClinicalTrials.gov from inception to July 23, 2024. An updated search was performed on August 23, 2025. The search strategy was refined for the update to adopt the latest MASLD/MASH nomenclature, ensuring optimal retrieval of recently published studies. The complete search strategies for all databases across both time points are detailed in Appendix 1 (initial search) and Appendix 2 (updated search).

### Selection criteria

The inclusion criteria for this analysis were formulated according to the PICOS framework. We included RCTs that enrolled adult patients (≥ 18 years old) with metabolic dysfunction-associated steatohepatitis (MASH, formerly known as NASH). Additionally, recognizing the shared pathophysiological pathways between metabolic diseases, cardiovascular diseases, and cancer [16–18], studies conducted in populations with obesity or T2DM that reported predefined liver injury biomarkers and metabolic parameters were also included for exploratory analysis. All trials were required to have a follow-up of at least 12 weeks with semaglutide at any dose, a comparator group receiving either placebo or active control, and must have reported outcomes assessing liver injury and safety. Editorials, letters, reviews, brief communications, and observational studies were excluded from consideration.

### Data extraction

Two authors (Ranran Kan and Siyi Wang) independently screened the records using EndNote X9 (Clarivate) to identify eligible studies. The extracted data included the trial name, first author, publication year, sample size, baseline characteristics of participants, details of the intervention and comparator (including formulation, dosage, and frequency), follow-up duration, and all relevant outcome data. The extracted outcomes included predefined hepatic parameters, cardiometabolic profiles, and safety assessments. Any discrepancies encountered during data extraction were resolved through discussion and consultation with a third author (Danpei Li) to achieve a consensus.

To ensure comparability and enable pooled analysis of semaglutide dosages across studies that employed different administration frequencies, all doses were converted to a weekly equivalent. For the trials utilizing daily subcutaneous administration, the daily dose was multiplied by seven to calculate the weekly equivalent (e.g., a daily dose of 0.2 mg was converted to 1.4 mg/week). This standardization was applied because the majority of the included trials used a once-weekly dosing regimen, and this approach allows for a consistent dose-response analysis across the entire dataset.

### Outcomes

#### Primary outcomes

The co-primary outcomes of this meta-analysis were the changes in gold-standard histological endpoints in patients with confirmed MASH: (1) MASH Resolution, defined an NAS of 0 for ballooning and 0 to 1 for inflammation; (2) Fibrosis Improvement, defined as at least a 1-stage reduction on the MASH CRN fibrosis scale [19].

#### Secondary outcomes

Secondary outcomes were comprehensive and included several key domains. Liver-related outcomes comprised non-invasive biomarkers of liver injury, specifically changes in Liver Steatosis assessed by magnetic resonance imaging proton density fat fraction (MRI-PDFF) [20], alterations in Liver Stiffness measured via magnetic resonance elastography (MRE) in kPa, as well as changes in the Fibrosis Test Score (FTS) and the enhanced liver fibrosis (ELF) score. Additionally, serum levels of the liver enzymes alanine aminotransferase (ALT), aspartate aminotransferase (AST), and gamma-glutamyl transferase (GGT) were evaluated. Metabolic parameters of interest covered changes in body weight control, lipid profile parameters and glycemic measures.

#### Safety outcomes

Safety outcomes encompassed the incidence of death, cardiovascular events, any-cause adverse events leading to treatment discontinuation, gastrointestinal events, as well as specific events including cholelithiasis, pancreatitis.

### Risk of bias and quality assessment

The methodological quality and risk of bias were evaluated using the Cochrane Risk of Bias tool for randomized trials (RoB 2.0) [21]. This tool enables a structured assessment across five key domains: (1) the randomization process; (2) deviations from the intended interventions; (3) missing outcome data; (4) measurement of the outcome and (5) selection of the reported result. Two reviewers independently performed the assessments, and any discrepancies were resolved through discussion or consultation with a third author to reach consensus.

The overall certainty of evidence for primary outcomes was assessed using the GRADE approach [22]. Evidence from randomized trials starts as high certainty but can be downgraded based on five domains: risk of bias, inconsistency, indirectness, imprecision, and publication bias. Any discrepancies were resolved by consensus.

### Data analysis and statistical methods

Continuous outcomes were summarized as weighted mean differences (WMDs) with 95% confidence intervals (CIs), while dichotomous outcomes as risk ratios (RRs) with 95% CIs. All meta-analyses were performed using the DerSimonian-Laird random-effects model to account for potential clinical and methodological heterogeneity among studies. The choice of this model considered the variation in dosing and study designs, while acknowledging its sensitivity to small-study effects given the wide range of trial sizes included. Heterogeneity was quantitatively assessed using the *I*^*2*^ statistic, with values greater than 50% indicating substantial heterogeneity, consistent with established guidelines [23].

Univariable meta-regression was performed to examine the association between prespecified covariates (including weekly dose, baseline BMI, study design, and intervention duration) and protective effects for key liver outcomes (containing at least ten studies). The adjusted R^2^ statistic was calculated to estimate the proportion of between-study variance explained by each covariate.

Predefined subgroup analyses were conducted to investigate potential sources of heterogeneity and evaluate the influence of semaglutide dosing and intervention duration. Subgroups were stratified by the following: weekly semaglutide dose (lower tertile: < 1.0 mg; mid tertile: 1.0–1.9 mg; upper tertile: ≥ 2.0 mg), intervention duration (short-term: < 12 months; long-term: ≥ 12 months), baseline BMI (< 35 vs. ≥ 35 kg/m^2^), baseline body weight (< 95 vs. ≥ 95 kg), study design (double-blind vs. open-label), control type (placebo vs. active drug), diagnostic method (biopsy vs. non-biopsy), and disease status (T2DM, MASH, or MASH with cirrhosis).

In our dose-response analysis, we used natural cubic splines to address potential non-linearities, chosen for their stable boundary estimates and reduced extrapolation bias in limited-dose studies. We placed knots at the 33rd and 67th percentiles (0.7 mg and 1.6 mg) to balance flexibility and overfitting. Model fit was assessed with the Akaike Information Criterion (AIC), and non-linearity was tested using a likelihood ratio test against a linear meta-regression.

Publication bias was assessed through visual inspection of funnel plots and Egger’s regression test for outcomes with at least ten studies. To evaluate the robustness of the findings, sensitivity analysis was performed by sequentially excluding individual studies and recalculating the pooled estimates. Additionally, to address potential clinical heterogeneity, we conducted sensitivity analyses restricted to studies enrolling exclusively patients with histologically confirmed MASH. For each liver outcome, we compared the results from the main analysis (including all eligible studies) with those from the sensitivity analysis (restricted to histologically confirmed MASH populations). Heterogeneity was reassessed using the *I*^*2*^ statistic within this subgroup. All statistical analyses were performed using STATA version 16.0 and RevMan version 5.4 software (Cochrane Collaboration, Oxford, UK).

## Results

### Search results and characteristics of studies

**Fig. 1:**
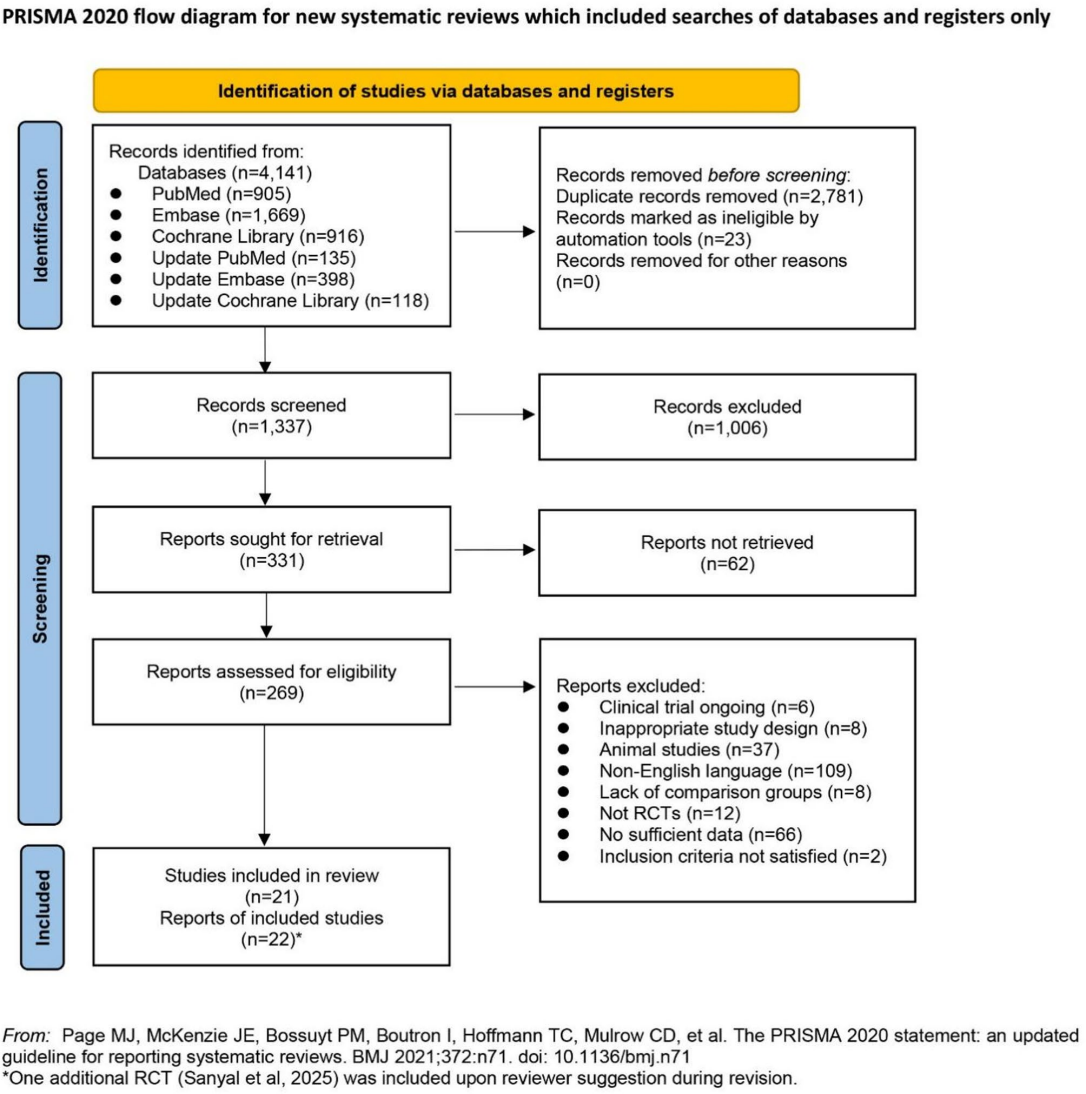
PRISMA Flow Diagram of Study Selection Process

A total of 4,141 records were identified through systematic database searches. After the removal of duplicates and initial screening, 22 RCTs [24–45] met the eligibility criteria and were included in the final meta-analysis (Fig. 1). The baseline characteristics of the included studies are summarized in Table 1. These trials enrolled a total of 32,013 participants, with 17,560 assigned to semaglutide groups and 14,453 to control groups. The intervention duration ranged from 12 to 104 weeks. Among the included trials, five utilized a daily dosing regimen [28, 33, 38, 39, 45], one trial incorporated both daily and weekly administration schedules [26], and the remaining sixteen employed weekly administration. For trials using daily subcutaneous semaglutide [28, 45], the doses were recalculated as weekly doses for further analysis. Furthermore, seven studies utilized an open-label design[24–26, 29, 31, 40, 42]. Among these, only one trial employed a placebo comparator [26], while the remaining six utilized active-control agents.

**Table 1:** Baseline characteristics of the included RCTs

Source	No. of patients	Masking	Study arms	Population	Mean age, y	Male, (%)	Mean BMI, kg/m^2^	Diabetes (%)	Semaglutide, mg	Comparator, mg	Trial duration, week	Outcomes
Ahmann 2018 [24]	809	Open-label	2	T2DM	56.6	55.3	33.8	100.0	1.0 s.c., Qw	exenatide ER 2.0 s.c., Qw	56	Metabolic Outcomes
Alkhouri 2022 [25]	108	Open-label	5	MASH (Biopsy)	54.8	45.4	34.8	-	2.4 s.c., Qw	*Combined therapy	35	Liver Stiffness, FTS, ELF, ALT, GGT, Metabolic Outcomes
Davies 2017 [26]	632	Open-label	9	T2DM	57.1	62.7	31.7	100.0	1.0 s.c., Qw 2.5, 5.0, 10.0, 20.0, 40.0 (oral), Qd	Placebo	26	Metabolic Outcomes
Davies 2021 [27]	1,210	Double-blind	3	Obesity and T2DM	55.0	49.1	35.7	100.0	1.0, 2.4 s.c., Qw	Placebo	68	ALT, AST, GGT, Metabolic Outcomes
Flint 2021 [28]	67	Double-blind	2	MASH (MRI)	60.0	70.1	30.0	73.0	0.4 s.c., Qd	Placebo	72	Liver Steatosis, Liver Stiffness, FTS, ALT, AST, GGT, Metabolic Outcomes
Frías 2021 [29]	1,879	Open-label	4	T2DM	56.6	47.0	34.2	100.0	1.0 s.c., Qw	tirzepatide 5.0, 10.0, 15.0 s.c., Qw	40	ALT, AST, Metabolic Outcomes
Garvey 2022 [30]	304	Double-blind	2	Obesity	47.3	22.4	38.5	0.0	2.4 s.c., Qw	Placebo	104	Metabolic Outcomes
Kimura 2023 [31]	108	Open-label	2	T2DM	62.7	29.5	29.3	100.0	1.0 s.c., Qw	dulaglutide 0.75 s.c., Qw	24	ALT, AST, GGT, Metabolic Outcomes
Knop 2023 [33]	667	Double-blind	2	Obesity	50.0	27.0	37.5	0.0	50.0 (oral), Qd	Placebo	68	Metabolic Outcomes
Lincoff 2023 [34]	17,604	Double-blind	2	Obesity with CVD	61.6	72.3	33.3	66.4% prediabetes	2.4 s.c., Qw	Placebo	104	Metabolic Outcomes
Loomba 2023 [35]	71	Double-blind	2	MASH related cirrhosis (Biopsy)	59.5	31.0	34.9	75.0	2.4 s.c., Qw	Placebo	48	MASH Resolution, Improvement of Fibrosis Stage, Liver Steatosis, Liver Stiffness, ALT, AST, GGT, Metabolic Outcomes
Marso 2016 [36]	3,297	Double-blind	4	T2DM	64.6	60.7	-	100.0	0.5, 1.0 s.c., Qw	Placebo	104	Metabolic Outcomes
Nauck 2016 [37]	415	Double-blind	9	T2DM	55.2	65.0	30.9	100.0	0.1, 0.2, 0.4, 0.8, 1.6 s.c., Qw	Placebo	12	ALT, AST, Metabolic Outcomes
Newsome 2021 [45]	320	Double-blind	4	MASH (Biopsy)	55.0	39.0	35.8	62.0	0.1, 0.2. 0.4 s.c., Qd	Placebo	72	MASH Resolution, Improvement of Fibrosis Stage, Liver Steatosis, Liver Stiffness, FTS, ELF, ALT, AST, Metabolic Outcomes
O’Neil 2018 [38]	957	Double-blind	9	Obesity	47.0	35.0	39.3	0.0	0.05, 0.1, 0.2. 0.3, 0.4 s.c., Qd	Placebo	52	Metabolic Outcomes
Pratley 2019 [39]	711	Double-blind	3	T2DM	56.0	52.0	33.0	100.0	14.0 (oral), Qd	Placebo	52	Metabolic Outcomes
Romero-Gómez 2023 [40]	145	Open-label	2	MASH (MRI)	49.5	55.2	34.3	33.1	1.0 s.c., Qw	efinopegdutide 10 s.c., Qw	32	Liver Steatosis, ELF, ALT, AST, Metabolic Outcomes
Rubino 2021 [41]	803	Double-blind	2	Obesity	46.0	21.0	38.4	0.0	2.4 s.c., Qw	Placebo	68	Metabolic Outcomes
Seino 2018 [42]	308	Open-label	3	T2DM	58.3	76.3	25.4	100.0	0.5, 1.0 s.c., Qw	sitagliptin 100 (oral), Qd	30	Metabolic Outcomes
Wadden 2021 [43]	611	Double-blind	2	Obesity	46.0	19.0	38.0	0.0	2.4 s.c., Qw	Placebo	68	Metabolic Outcomes
Wilding 2021 [44]	1,961	Double-blind	2	Obesity	46.5	25.9	37.9	42.8% for prediabetes	2.4 s.c., Qw	Placebo	68	Metabolic Outcomes
Sanyal 2025 [32]	800	Double-blind	2	MASH (Biopsy)	56.0	57.1	34.5	55.9	2.4 s.c., Qw	Placebo	72	MASH Resolution, Improvement of Fibrosis Stage, Liver Stiffness, ELF, ALT, AST, Metabolic Outcomes

*Combined therapy for NASH included cilofexor and firsocostat, a nonsteroidal farnesoid X receptor (FXR) agonist and an investigational liver-directed acetyl-CoA carboxylase (ACC) inhibitor respectively

T2DM, type 2 diabetes mellitus; MASH, metabolic dysfunction-associated steatohepatitis; MRI, magnetic resonance imaging; CVD, cardiovascular disease; NAS, non-alcoholic fatty liver disease activity score; FTS, fibrosis test score; ELF, enhanced liver fibrosis score; ALT, alanine aminotransferase; AST, aspartate transaminase; GGT, gamma glutamyl transferase

### Risk of bias assessment

**Fig. 2:**
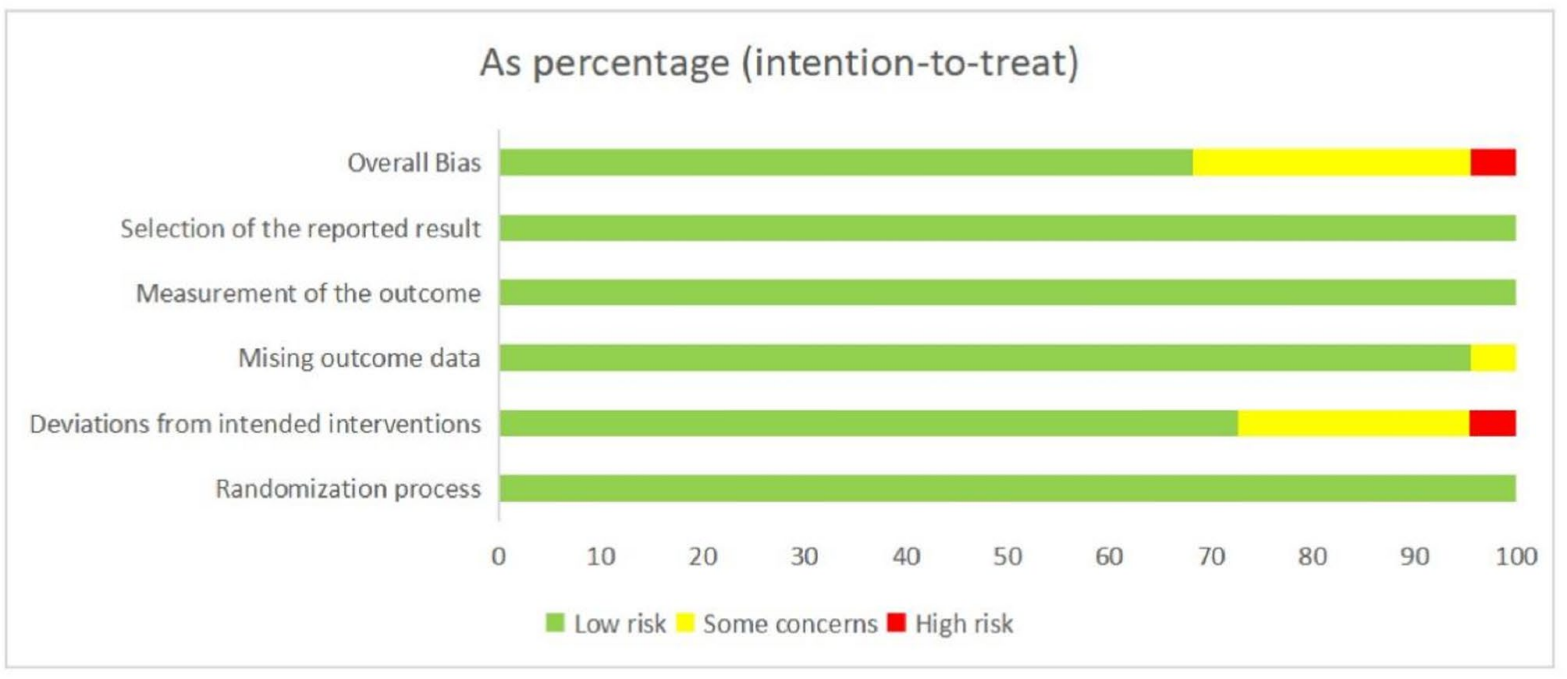
Summaries of the Risk of Bias (RoB) Assessment of the Included Studies

The risk of bias assessment for the included trials, performed using the Cochrane RoB 2.0 tool, is summarized in Fig. 2. Most studies were judged to be at low risk of bias across key domains, including the randomization process, measurement of outcomes, and selection of the reported result. A slightly higher prevalence of concerns was observed in the domain of “deviations from intended interventions”, which primarily due to the open-label design of several large-scale trials. The risk of bias due to missing outcome data was generally low across studies, with only a few trials raising some concerns in this area. Overall, the majority of studies were assessed as having a low risk of bias, supporting the robustness and validity of the meta-analytic results. Detailed study-level presentation is provided in Appendix 3.

### Meta-analysis of primary outcomes

**Fig. 3:**
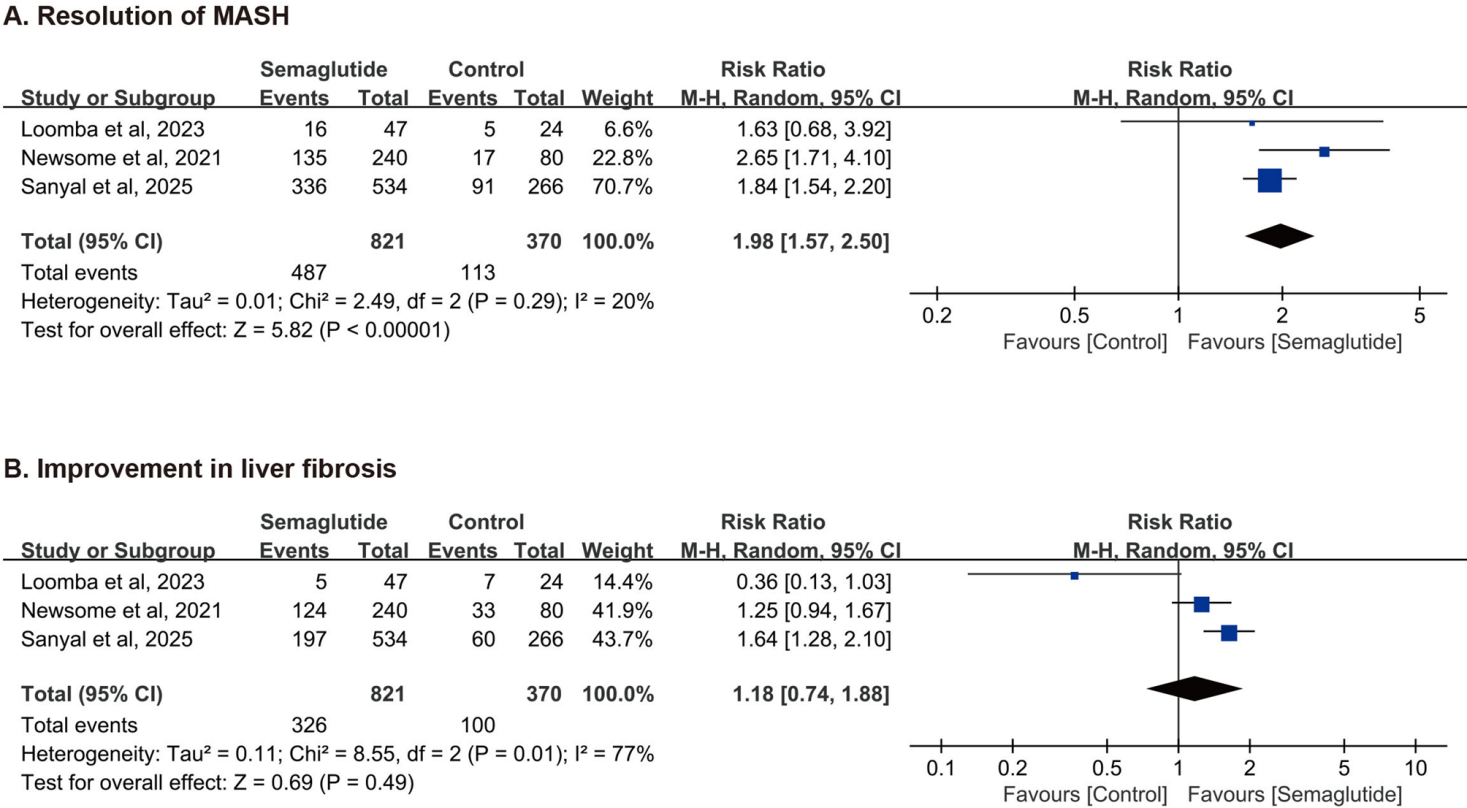
Forest Plot of Effects of Semaglutide on MASH Resolution (A) and Improvement in Fibrosis (B)

**Resolution of MASH. **Pooled analysis of three RCTs comprising 1,191 participants demonstrated that semaglutide was associated with a significantly higher rate of MASH resolution compared to control (RR = 1.98, 95%CI: 1.57 to 2.50, P < 0.00001; Fig. 3A). The heterogeneity among studies was low (*I*^2^= 20%, P = 0.29).

**Improvement in Liver Fibrosis.** In contrast, the pooled result for improvement in liver fibrosis (defined as ³ 1-stage reduction) did not reach statistical significance (RR = 1.18, 95%CI: 0.74 to 1.88, P = 0.49; Fig. 3B). Substantial heterogeneity was observed across the three trials for this outcome (*I*^2^= 77%, P = 0.01).

### Meta-analysis of liver-related secondary outcomes

#### Non-invasive biomarkers of liver pathology

**Fig. 4:**
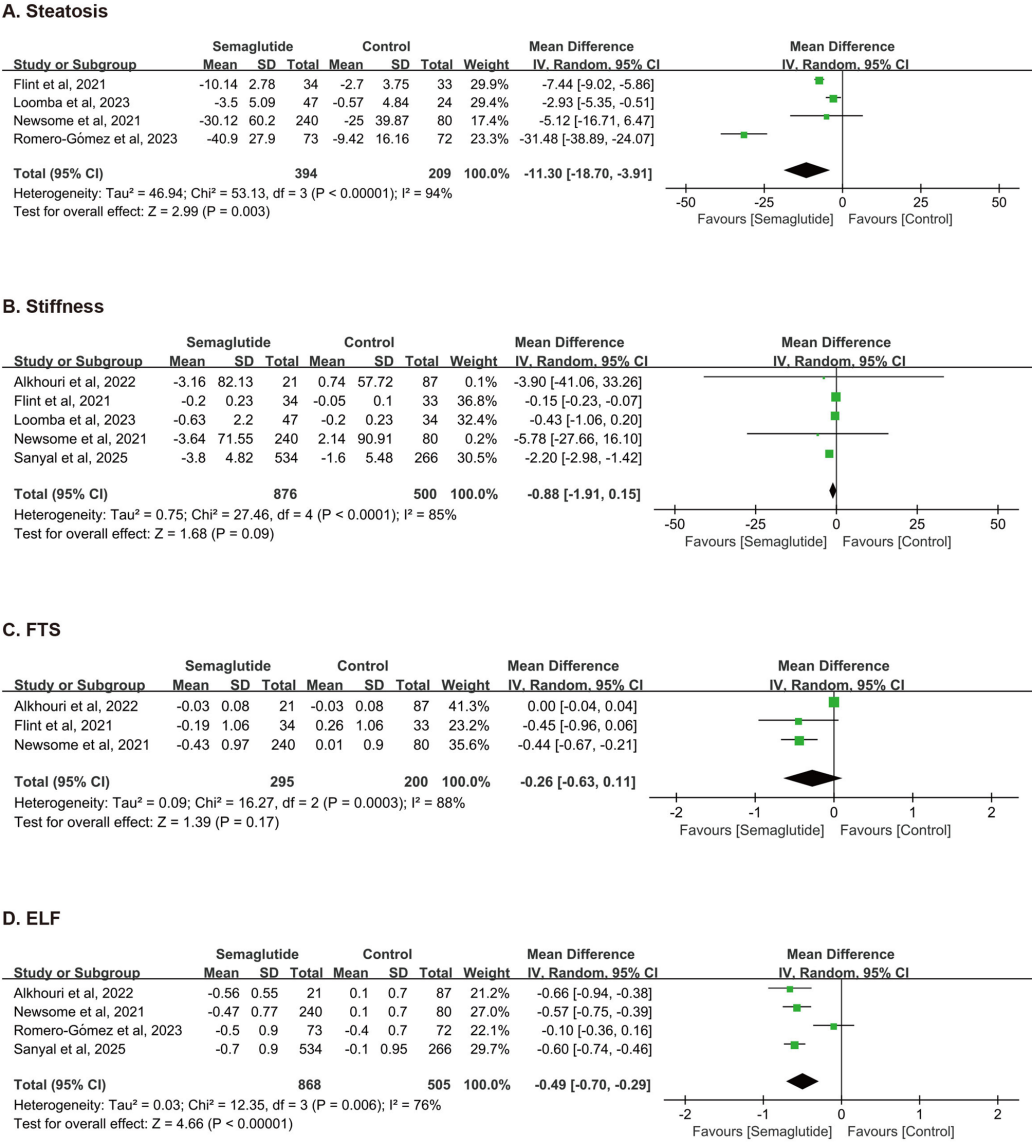
Forest Plot of Effects of Semaglutide on Liver Steatosis (A), Stiffness (B), FTS (C), and ELF (D). FTS, fibrosis test score; ELF, enhanced liver fibrosis score.

**Liver Steatosis** showed a significant reduction with semaglutide compared to control (WMD = −11.30%, 95%CI: −18.70 to −3.91, *P* = 0.003; Fig. 4A). However, substantial heterogeneity was observed among these studies (*I*^*2*^ = 94%, *P* < 0.00001). For **Liver Stiffness**, the pooled result did not show a statistically significant difference (WMD = −0.88 kPa, 95%CI: −1.91 to 0.15, *P* = 0.09; Fig. 4B), with high heterogeneity (*I*^*2*^ = 85%, *P* < 0.0001). Similarly, the **Fibrosis Test Score (FTS)** did not demonstrate a significant protective effect (WMD = −0.26, 95%CI: −0.63 to 0.11, *P* = 0.17; Fig. 4C), also with considerable heterogeneity (*I*^*2*^ = 88%, *P* = 0.0003). In contrast, the **Enhanced Liver Fibrosis (ELF)** score was significantly reduced in patients receiving semaglutide (WMD = −0.49, 95%CI: −0.70 to −0.29, *P* < 0.00001; Fig. 4D), with substantial heterogeneity (*I*^*2*^ = 76%, *P* = 0.006).

#### Liver enzymes

**Fig. 5:**
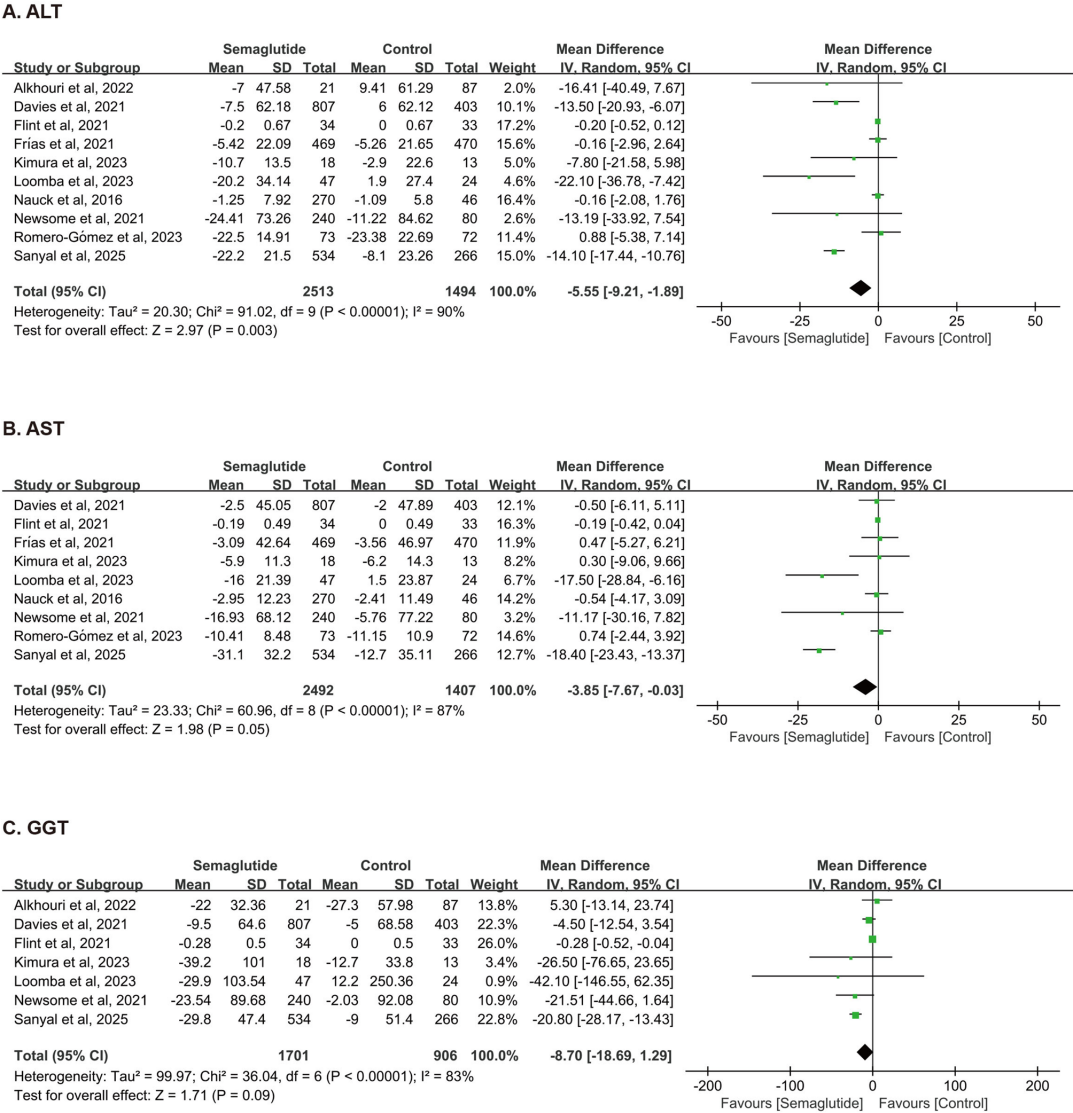
Forest Plot of Effects of Semaglutide on ALT (A), AST (B), and GGT (C). ALT, alanine aminotransferase; AST, aspartate transaminase; GGT, gamma-glutamyl transferase.

A total of ten studies involving 4,007 patients demonstrated a statistically significant reduction in ALT with semaglutide (WMD = −5.55 U/L, 95%CI: −9.21 to −1.89, *P* = 0.003; *I*^*2*^ *=* 90%; Fig. 5A). A significant change was observed in AST (WMD = −3.85 U/L, 95%CI: −7.67 to −0.03, *P* = 0.05; *I*^*2*^ *=* 87%; Fig. 5B). However, pooled analysis did not show a significant reduction in GGT levels (WMD = −8.70 U/L, 95%CI: −18.69 to 1.29, *P* = 0.09; *I*^*2*^ *=* 85%; Fig. 5C).

### Meta-regression analysis

To explore potential sources of heterogeneity, pre-specified meta-regression analyses were performed for ALT in Table 2, which had the largest number of available studies (*n* = 10). Weekly semaglutide dose emerged as a significant effect modifier (Coefficient: −4.14, 95%CI: −7.89 to −0.40; *P* = 0.032), accounting for 23.3% of the between-study variance. Higher baseline BMI was also significantly associated with greater reductions in ALT (Coefficient: −2.00, 95%CI: −5.06 to −1.06; *P* = 0.017), explaining 19.5% of the variance. Other covariates, including baseline body weight, mean age, blinding status, and control type, demonstrated minimal influence on heterogeneity (all *P* > 0.05; adjusted R^2^ < 6.5%). An exploratory meta-regression of AST, which included 9 studies, yielded consistent findings regarding the influence of weekly dose. Detailed results are provided in the Supplementary Table 1.

**Table 2:** Univariable Meta-Regression analyses exploring sources of heterogeneity for the change in ALT

Covariate	No. of studies	Coefficient	95% CI	*P*-value	Adjusted *R*^2^ (%)
Dose of semaglutide, mg/w	10	−4.14	(−7.89 to −0.40)	0.032*	23.31
Baseline BMI, kg/m^2^	10	−2.00	(−5.06 to −1.06)	0.017*	19.51
Baseline weight, kg	10	−0.47	(−1.51 to 0.57)	0.326	6.22
Mean age, y	10	−0.94	(−2.87 to 1.00)	0.296	2.91
Double-blind (Yes vs. No)	10	−5.71	(−18.19 to 6.78)	0.323	1.83
Active-control (Yes vs. No)	10	5.71	(−6.78 to 18.19)	0.323	1.83
Baseline HbA_1c_, %	10	3.02	(−5.08 to 11.11)	0.415	−2.95
Baseline FPG, mmol/L	10	1.46	(−5.28 to 8.20)	0.637	−13.14
Duration of intervention, w	10	−0.08	(−0.37 to 0.21)	0.622	−11.98
Sample size	10	< 0.01	(−0.01 to 0.01)	0.870	−21.49

ALT, alanine aminotransferase; CI, confidence interval; BMI, body mass index; HbA1c, glycated hemoglobin; FPG, fasting plasma glucose

The dependent variable for all analyses was the mean difference in ALT

Coefficient represents the change in the ALT mean difference per unit increase in the covariate. A negative coefficient indicates a greater reduction in ALT is associated with higher values of the covariate

*P < 0.05, ** *P* < 0.01, *** *P* < 0.001, **** *P* < 0.0001

### Subgroup analysis

**Fig. 6:**
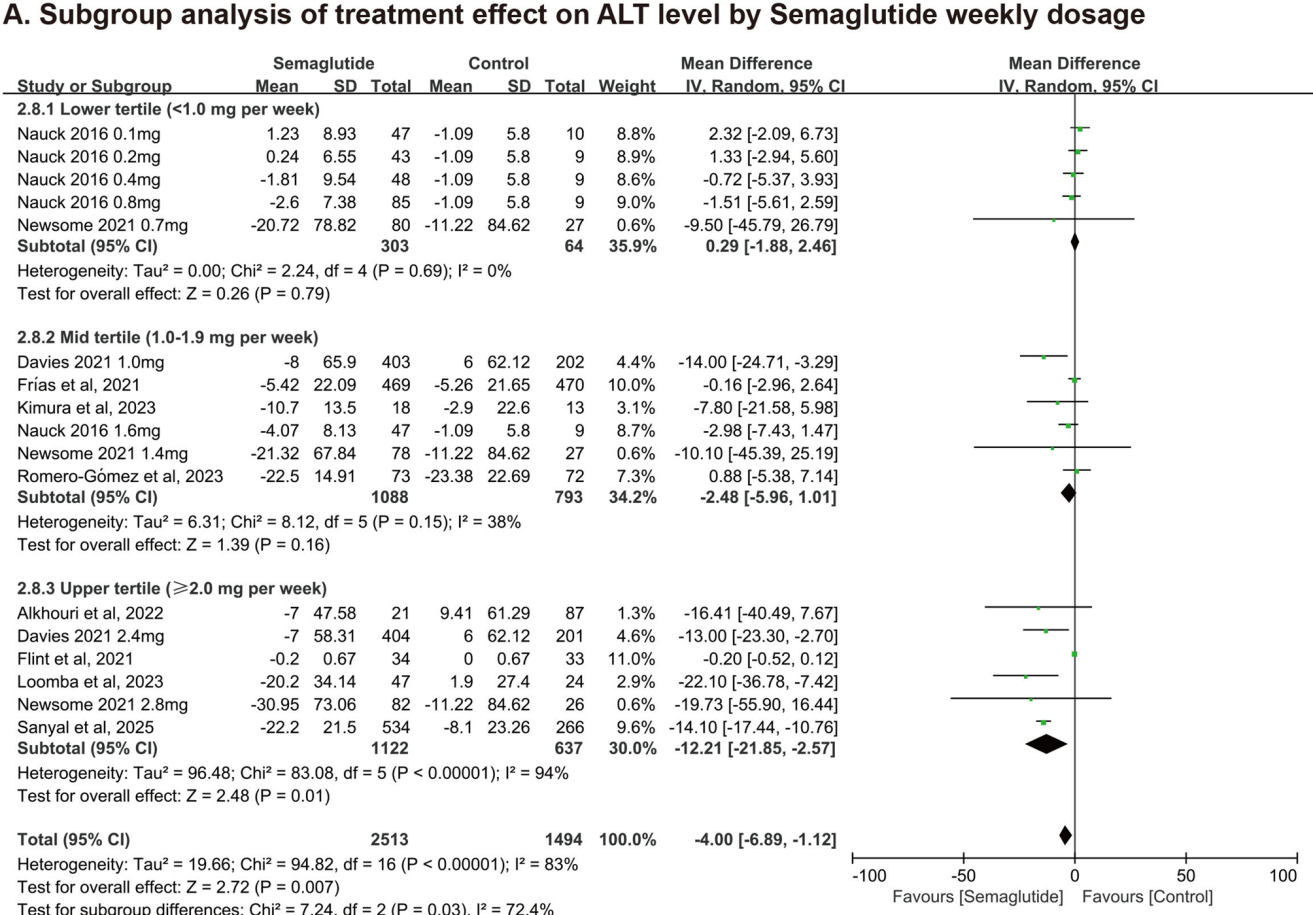
Subgroup Analysis of the Reduction in ALT Comparing Semaglutide and Controls Categorized by the Weekly Dosage. ALT, alanine aminotransferase.

Pre-specified subgroup analysis by weekly dosage demonstrated a potential dose-response trend (Fig. 6). The highest efficacy was observed in the high-dose group (≥ 2.0 mg/week: WMD = −12.21 U/L, 95%CI: −21.85 to −2.57; *P* = 0.01), followed by a modest and non-significant reduction in the mid-dose group (1.0–1.9 mg/week: WMD = −2.48 U/L, *P* = 0.16). The low-dose group (< 1.0 mg/week) showed negligible change (WMD = 0.29 U/L, *P* = 0.79). The test for subgroup differences was statistically significant (*P* = 0.03), supporting a significant monotonic dose-response relationship

Additional subgroup analyses were performed to investigate potential sources of heterogeneity and identify clinically relevant effect modifiers (Table 3). Greater ALT reductions were observed in participants with higher baseline BMI (≥ 35 kg/m^2^; WMD = −15.06 U/L) or body weight (≥ 95 kg; WMD = −11.62 U/L). Trial design influenced outcomes, with double-blind, placebo-controlled trials showing stronger protective effects (WMD = −7.93 U/L) than open-label or active-comparator studies. The most pronounced and homogeneous effect was observed in the subgroup of trials with biopsy-confirmed MASH patients (WMD = −14.50 U/L; *I*^*2*^ = 0%). Furthermore, longer intervention duration (≥ 12 months) was associated with a markedly greater reduction in ALT (WMD = −11.61 U/L) compared to shorter-term studies. Although an exploratory analysis suggested a potential trend toward greater efficacy with increasing disease severity, the finding for the MASH-cirrhosis subgroup (based on a single study [35]) must be interpreted with extreme caution.

**Table 3:** Subgroup analyses of hepatoprotective effect on ALT level

Subgroups	No. of studies	Effect estimate (95% CI)	I^2^		Test for subgroup differences
Overall effect	10	−5.55 (−9.21 to −1.89)	90%		
By baseline BMI					
BMI < 35	7	−3.33 (−7.01 to 0.35)	91%	Chi^2^ = 9.90, df = 1 (***P = 0.002***)	
BMI ≥ 35	3	−15.06 (−21.38 to −8.75)	0%		
By baseline weight					
Body weight < 95 kg	4	−0.20 (−0.52 to 0.11)	0%	Chi^2^ = 9.42, df = 1 (***P = 0.002***)	
Body weight ≥ 95 kg	6	−11.62 (−18.92 to −4.34)	75%		
By intervention duration					
Short-term (< 12 m)	5	−0.26 (−1.78 to 1.27)	0%	Chi^2^ = 5.15, df = 1 (***P = 0.02***)	
Long-term (≥ 12 m)	5	−11.61 (−21.30 to −1.93)	95%		
By study design					
Double-blind	6	−7.93 (−13.14 to −2.72)	94%	Chi^2^ = 6.48, df = 1 (***P = 0.01***)	
Open-label	4	−0.42 (−2.92 to 2.08)	0%		
By control group					
Placebo	6	−7.93 (−13.14 to −2.72)	94%	Chi^2^ = 6.48, df = 1 (***P = 0.01***)	
Active control	4	−0.42 (−2.92 to 2.08)	0%		
By disease progression					
T2DM (high-risk)	4	−3.30 (−7.69 to 1.10)	77%	Chi^2^ = 5.91, df = 2 (***P = 0.05***)	
MASH	5	−6.60 (−15.43 to 2.22)	94%		
MASH-cirrhosis	1*	−22.10 (−36.78 to −7.42)	Not applicable		
By MASH diagnosis**					
By biopsy	4	−14.50 (−17.68 to −11.31)	0%	Chi^2^ = 76.57, df = 1 (***P < 0.00001***)	
Non-biopsy	2	−0.20 (−17.24 to −0.38)	0%		

Data are presented as weighted mean difference (WMD) with 95% confidence interval (CI). Significant results (*P* < 0.05) are highlighted in bold

*This subgroup analysis is considered exploratory due to the inclusion of only one study within this category; the findings are hypothesis-generating and require validation in future research

** This subgroup includes only trials where all participants had biopsy-confirmed MASH, thereby reducing diagnostic heterogeneity and focusing on the core population of interest

CI, confidence interval; df, degrees of freedom

### Dose-response analysis

**Fig. 7:**
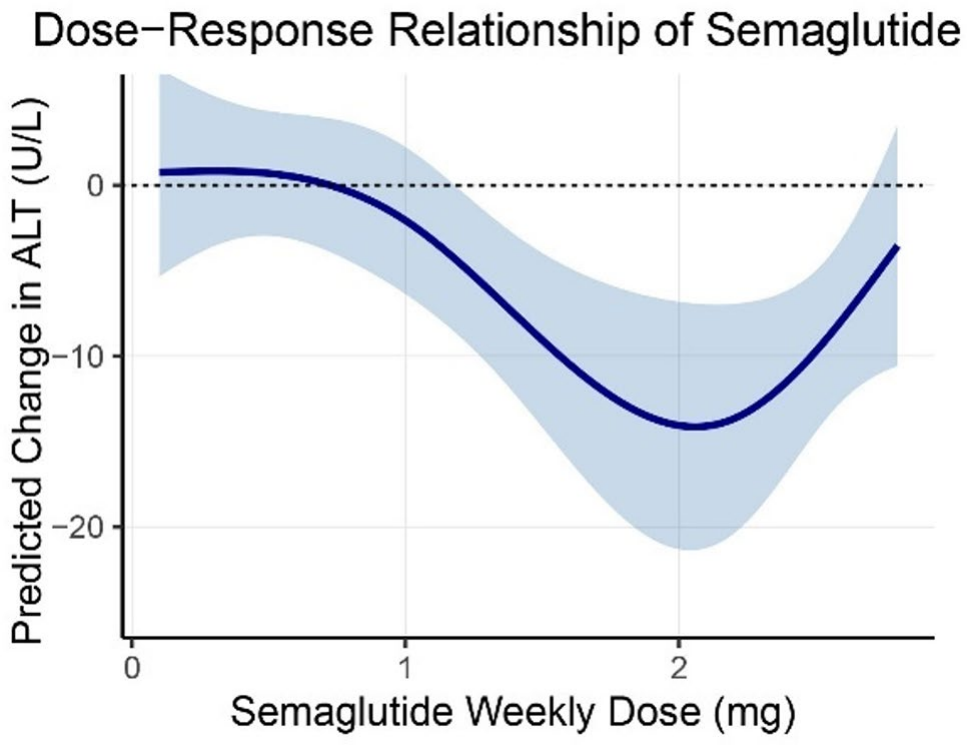
Non-Linear Lose-Response Relationship between Semaglutide Weekly Dose and ALT Reduction Modeled Using Natural Cubic Splines

To elucidate the dose-response relationship between semaglutide and ALT reduction, we compared a linear meta-regression model with a natural cubic spline model. The spline model demonstrated a substantially better fit, as indicated by a lower Akaike Information Criterion (AIC = 97.1 vs. 108.1) and a higher log-likelihood (logLik = −43.5 vs. −51.0). A likelihood ratio test confirmed significant non-linearity (LRT = 15.0, *P* = 0.0006), revealing that the association is not strictly linear but follows a complex pattern (Fig. 7)

Model-based predictions at specific doses showed minimal ALT reduction at lower doses (0.5 mg: −0.69 U/L, 95%CI: −2.96 to 4.34; 1.0 mg: −2.06 U/L, 95%CI: −6.37 to 2.24). The effect became more pronounced at intermediate doses, with the maximum reduction observed at 2.4 mg (−11.4 U/L, 95%CI: −16.7 to −6.1). At higher doses (2.8 mg), the benefit plateaued (−3.54 U/L, 95%CI: −10.6 to 3.5), suggesting that the optimal effect is achieved around 2.4 mg without further improvement beyond this point. These findings indicate a non-linear association, with the most substantial benefit occurring at moderate doses.

### Meta-analysis of metabolic parameters

**Weight Loss. **Semaglutide was associated with a significant reduction in **body weight** (WMD = −6.28 kg, 95%CI: −9.01 to −3.55; Fig. S1A). Patients also exhibited notable decreases in **BMI** (WMD = −2.80, 95%CI: −4.10 to −1.51; Fig. S1B) and **waist circumference** (WMD = −6.44 cm, 95%CI: −7.82 to −5.06; Fig. S1C).

**Glycemic Control.** Semaglutide administration resulted in statistically significant reductions in **FPG** (WMD = −0.41 mmol/L, 95%CI: −0.74 to −0.09; Fig. S2A) and **HbA**_**1c**_ (WMD = −0.53%, 95%CI: −0.78 to −0.29; Fig. S2B).

**Lipid Profiles.** Semaglutide was associated with favorable changes in serum lipid concentrations (Fig. S3-S4), including significant reductions in **TG** (WMD = −0.53 mmol/L, *P* = 0.01), **LDL** (WMD = −0.03 mmol/L, *P* < 0.00001), and **VLDL** (WMD = −0.55 mmol/L, *P* = 0.01), as well as a significant increase in **HDL** (WMD = 0.67 mmol/L, *P* < 0.00001). No significant changes were observed in **TC** (WMD = −0.44 mmol/L, *P* = 0.49) or **FFA** (WMD = −0.01 mmol/L, *P* = 0.93).

**Blood Pressure.** Semaglutide led to modest but significant reductions in both **SBP** (WMD = −3.57 mmHg, 95%CI: −4.70 to −2.44, Fig. S5A) and **DBP** (WMD = −0.87 mmHg, 95%CI: −1.39 to −0.34, Fig. S5B).

### Safety and adverse events

**Treatment Discontinuation**. The incidence of treatment discontinuation due to adverse events was 12.17% in the semaglutide group compared to 6.94% in the control group (RR = 1.60, 95%CI: 1.27 to 2.02, *P* < 0.0001; Fig. S5C).

**Mortality**. Semaglutide was associated with a significantly lower risk of all-cause mortality compared to control (RR = 0.82, 95%CI: 0.74 to 0.91, *P* = 0.0002; Fig. S6A), with no heterogeneity among studies (*I*^*2*^ = 0%). However, management with semaglutide was associated with a slightly higher risk of any **Adverse Events** (RR = 1.05, 95%CI: 1.01 to 1.09, *P* = 0.006; Fig. S6B), though with substantial heterogeneity (*I*^*2*^ = 82%). Furthermore, the risk of **Cardiovascular Disorders** was significantly reduced in the semaglutide group (RR = 0.83, 95%CI: 0.75 to 0.92, *P* = 0.0006; Fig. S6C), with low heterogeneity (*I*^*2*^ = 27%).

As expected, **Gastrointestinal Disorders** occurred more frequently with semaglutide than control (RR = 2.11, 95%CI: 1.61 to 2.77, *P* < 0.00001; Fig. S7A). A small but significant increase was also observed in the risk of **Gallbladder-Related Disorders** (RR = 1.28, 95%CI: 1.01 to 1.63, *P* = 0.04; Fig. S7B). However, no statistically significant difference was found in the incidence of **Pancreatitis** between the semaglutide and control groups (RR = 0.79, 95%CI: 0.52 to 1.21, *P* = 0.28; Fig. S7C), with no heterogeneity (*I*^*2*^ = 0%).

Subgroup analyses based on weekly dose and intervention duration revealed consistent safety outcomes for semaglutide, with no significant differences across subgroups (all *P* > 0.05). However, trends showed a dose-dependent increase in gastrointestinal disorder risk (RR ranging from 1.52 to 2.70) and greater cardiovascular protection at higher doses (RR = 0.76). Long-term use (≥ 12 months) led to a notable reduction in all-cause mortality (RR = 0.82) and cardiovascular events (RR = 0.83) without increasing discontinuation rates compared to short-term use.

### Sensitivity analyses

Leave-one-out sensitivity analysis was conducted to evaluate the robustness of the pooled estimates for key liver-related outcomes (Fig. S8). The results demonstrated that the significance and direction of the overall effects remained largely consistent across most endpoints.

Notably, the estimate for Improvement in Fibrosis was sensitive to the exclusion of the study by Loomba et al. (2023) [35]. When this trial was omitted, the summary effect size increased and the CI narrowed, indicating that its inclusion had a moderating influence on the overall pooled estimate. Conversely, the result for Liver Steatosis was sensitive to the exclusion of Flint et al. (2021) [28]. Removal of this study led to a substantial decrease in the point estimate, rendering the result statistically non-significant. Additionally, the result for Liver Stiffness was sensitive to the exclusion of Sanyal et al. (2025) [32], with a more pronounced improvement observed after omitting this trial.

No other outcome, including MASH Resolution, serum-based fibrosis markers (FTS and ELF), liver enzymes (ALT, AST, GGT), showed substantial changes in effect size or direction upon the removal of any single study. The stability of these results reinforces the reliability of the meta-analytic findings for these endpoints.

To address potential clinical heterogeneity arising from variations in patient populations, we performed sensitivity analyses restricted to trials with histologically confirmed MASH patients (Supplementary Table S5). Restricting the analysis to this homogeneous subgroup substantially reduced heterogeneity for several outcomes. Notably, for liver steatosis, ALT, AST, and ELF, the *I*^*2*^ values decreased to 0%, indicating that the high heterogeneity observed in the main analyses was largely attributable to differences in diagnostic confirmation across studies. The effect estimates remained consistent in direction for most outcomes, supporting the robustness of the primary findings.

### Publication bias

Publication bias was assessed using funnel plots and Egger’s regression test for meta-analyses containing more than ten studies (Fig. S9-S11). Visual inspection revealed generally symmetrical funnel plots for most outcomes, suggesting a low risk of substantial publication bias. This was corroborated statistically by Egger’s tests, which were non-significant for all outcomes except the incidence of adverse events (*P* = 0.016; Fig. S11A). The presence of potential small-study effects for adverse events is likely attributable to their high and variable incidence rates across trials of differing sizes and designs, rather than to selective publication.

### Certainty of evidence (GRADE)

The overall certainty of evidence for the primary liver-related outcomes was assessed using the GRADE approach (Appendix 4). For the critical outcome of MASH Resolution, the evidence was rated as high certainty, indicating strong confidence that the estimated effect is close to the true effect. Similarly, the evidence for the reduction in ALT levels was also assessed as high certainty, supported by a clear dose-response gradient and a strong association.

For Improvement in Fibrosis, the certainty was moderate, downgraded one level due to substantial heterogeneity. The evidence for Liver Steatosis was rated as moderate certainty, downgraded for risk of bias and heterogeneity. Similarly, evidence for Liver Stiffness and the ELF score was of moderate certainty, each downgraded one level for risk of bias. However, the evidence for the FTS was assessed as low certainty, downgraded twice due to risk of bias and considerable heterogeneity.

## Discussion

 This systematic review and meta-analysis provide comprehensive evidence that semaglutide confers significant benefits across multiple clinical domains in patients with MASH. The therapy is associated with meaningful improvements in key histological endpoints, non-invasive biomarkers of liver injury, and cardiometabolic parameters. A central finding of this analysis is that the therapeutic efficacy of semaglutide is substantially influenced by semaglutide regimen and baseline patient characteristics. Our dose-response analysis further revealed a non-linear relationship, with the most pronounced effects observed at doses around 2.4 mg, indicating an optimal range for ALT reduction. Additionally, longer intervention durations and higher baseline BMI were associated with greater benefits. The safety profile was consistent with the known class effects of GLP-1 RAs. This synthesis offers a timely and evidence-based overview of semaglutide’s multifaceted role in the management of MASH and its associated metabolic comorbidities, providing crucial insights for clinical practice and future research.

By incorporating data from the recently published trial by Sanyal et al. [32], this meta-analysis provides the first pooled evaluation of semaglutide’s effects on gold-standard histological outcomes in MASH, demonstrating a robust promotion of MASH Resolution despite the absence of significant improvement in Fibrosis Regression. This discrepancy may stem from the relatively short intervention duration in many included trials, which is likely insufficient to reverse established fibrotic lesions. These findings are consistent with multiple studies [32, 45, 46], its capacity to reverse liver fibrosis remains limited and appears less prominent compared with other therapeutic regimens [47]. Consequently, further research is essential to clarify semaglutide’s potential role in fibrosis modification, particularly through exploration of combination therapies with antifibrotic agents.

The differential effects of semaglutide on the resolution of MASH compared to fibrosis regression warrant further scholarly examination. Semaglutide exhibits a significant capacity to resolve steatohepatitis, largely attributable to its substantial metabolic advantages, anti-inflammatory properties, and direct benefits to hepatocyte health. However, its efficacy in addressing established fibrosis appears limited. This discrepancy aligns with the current pathophysiological understanding that fibrosis regression requires not only the removal of injurious triggers (which semaglutide effectively provides) but also the active degradation of the collagenous scar tissue itself [48]. The mechanisms underlying fibrosis resolution, such as reprogramming or apoptosis of activated hepatic stellate cells, may not be sufficiently targeted by GLP-1 RA alone [49]. This understanding underscores the need to investigate combination therapies. Co-administration of semaglutide with agents possessing direct anti-fibrotic activity (e.g., FXR agonists, ASK1 inhibitors, or monoclonal antibodies against pro-fibrotic pathways) represents a promising strategic direction [50].

Our investigation into heterogeneity revealed that the hepatoprotective effect of semaglutide was modified by several potential factors, with enhanced efficacy observed in patients with higher baseline BMI and body weight, underscoring its role in addressing metabolic drivers of MASH. In addition, previous study has shown that in patients with T2DM, the effect of semaglutide is slightly lower than that in patients without diabetes [51], indicating that individualized management strategies may enhance the therapeutic effect of semaglutide. The consistency of effects was also affected by trial design, with the most consistent effects seen in double-blind, placebo-controlled trials involving biopsy-confirmed MASH patients, highlighting the importance of rigorous phenotyping and potential biases in open-label studies. The sensitivity analyses restricted to histologically confirmed MASH populations revealed that clinical heterogeneity in diagnostic methods contributed significantly to the statistical heterogeneity observed in several outcomes. This underscores the importance of standardized, biopsy-based patient selection in future trials to yield more precise estimates of treatment effects.

The inclusion of patients with T2DM or obesity without confirmed MASH improves generalizability but demands outcome-specific interpretation. Histologic benefits (particularly MASH resolution) are supported primarily by evidence from biopsy-confirmed MASH populations, limiting their extrapolation to broader metabolic cohorts. Conversely, metabolic improvements were consistent across all patient types, suggesting broader applicability. For biomarkers like ALT, our preliminary exploratory subgroup analyses provide crucial distinction: a significant reduction was evident in the overall pool, but the effect was markedly stronger and highly homogeneous in trials enrolling biopsy-confirmed MASH patients (WMD = −14.50 U/L; I² = 0%) compared to those with high-risk conditions like T2DM (WMD = −3.30 U/L; I² = 77%; P for interaction < 0.00001). This gradient of response, from general metabolic improvement to specific liver injury reduction, underscores that the clinical implications of semaglutide are profoundly outcome-dependent and must be interpreted within the context of the underlying patient population. Further refinement by fibrosis stage or exploration of oral semaglutide was constrained by limited data, underscoring the need for future targeted studies.

Laboratory parameters, including liver enzymes and serum lipid profiles, provide readily accessible and clinically useful biomarkers, although they are not direct surrogates for histological activity in MASH. While these markers do not fully capture core pathological features such as lobular inflammation or fibrosis, they offer valuable complementary information regarding systemic metabolic improvements and hepatobiliary injury. Previous studies have indicated that elevated liver enzyme levels are closely associated with disease progression in MASH [52]. Additionally, dyslipidemia, particularly hypertriglyceridemia, has been recognized as an independent predictor of MASH [53]. The inclusion of these laboratory parameters as secondary or exploratory endpoints enables a more comprehensive evaluation of semaglutide’s multifaceted effects and aligns well with real-world clinical monitoring practices.

The comprehensive safety profile of semaglutide offers a balanced perspective on its clinical use. Although semaglutide is associated with significant cardioprotective effects and a reduction in all-cause mortality, it also leads to a higher incidence of gastrointestinal adverse events, as well as an increased risk of gallbladder-related disorders. These findings emphasize the importance of patient counseling, careful monitoring during therapy initiation, and long-term vigilance in clinical practice. Overall, the risk-benefit profile supports semaglutide as a safe and effective therapeutic option when adverse effects are proactively managed.

## Limitations

Several limitations may affect the results of our study. Firstly, significant heterogeneity was observed across included studies, which likely stems from differences in patient populations, such as the prevalence of diabetes and obesity, diagnostic criteria, and trial design. Additionally, the substantial disparity in the sizes of the included trials (ranging from approximately 100 to over 17,000 participants) introduces a potential for small-study bias. Although a random-effects model was employed, this bias may persist if smaller studies report systematically larger effect sizes than larger, potentially more robust trials. Although subgroup and meta-regression analyses were conducted, considerable heterogeneity persisted for some outcomes, suggesting that pooled estimates should be interpreted with caution. Secondly, the limited number of trials and variations in dosing regimens also restricted the precision of dose-response analyses. Notably, the conversion of daily to weekly dosing equivalents, while necessary for harmonization, may not fully reflect pharmacokinetic differences and thus could introduce misclassification. Thirdly, although the overall risk of bias was generally low, some concerns were identified, particularly regarding deviations from intended interventions in open-label trials. Furthermore, the GRADE assessment indicated that the certainty of evidence was from moderate to low for several outcomes, due largely to imprecision and study design limitations. Finally, the inclusion of trials involving patients with obesity or T2DM, though hypothesis-generating and pathophysiological relevant, introduces clinical heterogeneity that may affect the generalizability of findings to dedicated MASH populations. Further large-scale, double-blind trials with longer follow-up and more homogeneous patient cohorts are needed to confirm these results and better establish the optimal intervention duration and dosing strategy for semaglutide in MASH.

## Conclusion

Semaglutide represents a promising pharmacotherapeutic option for MASH, demonstrating significant improvements in histologic resolution, liver injury biomarkers, and metabolic parameters, particularly at higher doses and longer intervention durations, though its effect on fibrosis regression remains limited.

## Supplementary Information

Supplementary Material 1

Supplementary Material 2

Supplementary Material 3

Supplementary Material 4

Supplementary Material 5

## Data Availability

All data supporting the findings of this study are available in the manuscript and supplementary materials. The full set of RevMan files used for meta-analyses has been deposited in Zenodo (DOI: 10.5281/zenodo.16992817).
