# Supplementary material for: The impact of semaglutide on liver outcomes in patients with or at risk of MASH: a dose and duration response meta-analysis of randomized trials
Source: Diabetol Metab Syndr. 2025 Nov 24;17:439. doi: 10.1186/s13098-025-01995-z (PMC12642090; doi:10.1186/s13098-025-01995-z)
Supplement: Supplementary file 1 — Supplementary Material 1 [file 13098_2025_1995_MOESM1_ESM.docx]

**Appendix 1. Initial Search Strategies for All Databases.**

Pubmed: 905 Results

Date of search: July 23, 2024

(((semaglutide) OR (rybelsus) OR (Ozempic)) AND ((non-alcoholic steatohepatitis) OR (Non-alcoholic Fatty Liver Disease) OR (NAFLD) OR (Nonalcoholic Fatty Liver Disease) OR (Fatty Liver, Nonalcoholic) OR (Fatty Livers, Nonalcoholic) OR (Liver, Nonalcoholic Fatty) OR (Livers, Nonalcoholic Fatty) OR (Nonalcoholic Fatty Liver) OR (Nonalcoholic Fatty Livers) OR (Nonalcoholic Steatohepatitis) OR (Nonalcoholic Steatohepatitides) OR (Steatohepatitides, Nonalcoholic) OR (Steatohepatitis, Nonalcoholic))) AND (((randomized controlled trial [Publication Type]) OR (controlled clinical trial [Publication Type]) OR (randomized [Title/Abstract]) OR (randomised [Title/Abstract]) OR (placebo [Title/Abstract]) OR (drug therapy [MeSH Subheading]) OR (randomly [Title/Abstract]) OR (trial [Title/Abstract])) NOT ((animals[MeSH Terms]) NOT (humans[MeSH Terms])))

Embase: 1,669 results

Date of search: July 23, 2024

1 (semaglutide or rybelsus or Ozempic).af. 4,159

2 (non-alcoholic steatohepatitis or Non-alcoholic Fatty Liver Disease or NAFLD or Nonalcoholic Fatty Liver Disease or Fatty Liver, Nonalcoholic or Fatty Livers, Nonalcoholic or Liver, Nonalcoholic Fatty or Livers, Nonalcoholic Fatty or Nonalcoholic Fatty Liver or Nonalcoholic Fatty Livers or Nonalcoholic Steatohepatitis or Nonalcoholic Steatohepatitides or Steatohepatitides, Nonalcoholic or Steatohepatitis, Nonalcoholic).af. 80,839

3 randomized.ab,ti. 970,981

4 randomised.ab,ti. 193,676

5 placebo.ab,ti. 366,414

6 randomly.ab,ti. 556,992

7 trial.ab,ti. 1,122,384

8 drug therapy.sh. 942,954

9 randomized controlled trial.pt,sh. 786,697

10 controlled clinical trial.pt,sh. 471,228

11 3 or 4 or 5 or 6 or 7 or 8 or 9 or 10 3,199,680

12 1 and 2 and 11 1,669

Cochrane Library: 916 results

Date of search: July 23, 2024

#1 (semaglutide):ti,ab,kw OR (rybelsus):ti,ab,kw OR (Ozempic):ti,ab,kw 1,015

#2 semaglutide 1,027

#3 #1 or #2 1,028

#4 non-alcoholic steatohepatitis 1,268

#5 (Non-alcoholic Fatty Liver Disease):ti,ab,kw OR (NAFLD):ti,ab,kw OR (Nonalcoholic Fatty Liver Disease):ti,ab,kw OR (Fatty Liver, Nonalcoholic):ti,ab,kw OR (Fatty Livers, Nonalcoholic):ti,ab,kw 4,355

#6 (Liver, Nonalcoholic Fatty):ti,ab,kw OR (Livers, Nonalcoholic Fatty):ti,ab,kw OR (Nonalcoholic Fatty Liver):ti,ab,kw OR (Nonalcoholic Fatty Livers):ti,ab,kw OR (Nonalcoholic Steatohepatitis):ti,ab,kw 4,559

#7 (Nonalcoholic Steatohepatitides):ti,ab,kw OR (Steatohepatitides, Nonalcoholic):ti,ab,kw OR (Steatohepatitis, Nonalcoholic):ti,ab,kw 1,728

#8 #4 or #5 or #6 or #7 4,816

#9 #3 and #8 in Trials 916

**Appendix 2. Updated Search Strategies for All Databases.**

Pubmed: 135 Results

Date of search: August 23, 2025

#1 (semaglutide) OR (rybelsus) OR (Ozempic) 3,455

#2 (non-alcoholic steatohepatitis) OR (Non-alcoholic Fatty Liver Disease) OR (NAFLD) OR (Nonalcoholic Fatty Liver Disease) OR (Fatty Liver, Nonalcoholic) OR (Fatty Livers, Nonalcoholic) OR (Liver, Nonalcoholic Fatty) OR (Livers, Nonalcoholic Fatty) OR (Nonalcoholic Fatty Liver) OR (Nonalcoholic Fatty Livers) OR (Nonalcoholic Steatohepatitis) OR (Nonalcoholic Steatohepatitides) OR (Steatohepatitides, Nonalcoholic) OR (Steatohepatitis, Nonalcoholic) 52,707

#3 (Metabolic Associated Fatty Liver Disease) OR (MAFLD) OR (Metabolic Dysfunction-associated Steatotic Liver Disease) OR (MASLD) OR (Metabolic Associated Steatohepatitis) OR (MASH) 42,048

#4 (randomized controlled trial [Publication Type]) OR (controlled clinical trial [Publication Type]) OR (randomized [Title/Abstract]) OR (randomised [Title/Abstract]) OR (placebo [Title/Abstract]) OR (drug therapy [MeSH Subheading]) OR (randomly [Title/Abstract]) OR (trial [Title/Abstract]) 4,302,983

#5 (animals [MeSH Terms]) NOT (humans [MeSH Terms]) 5,365,169

#6 (#1 AND (#2 OR #3) AND #4) NOT #5 135

Embase: 398 results

Date of search: August 23, 2025

1 (semaglutide or rybelsus or Ozempic).af. 9,424

2 (non-alcoholic steatohepatitis or Non-alcoholic Fatty Liver Disease or NAFLD or Nonalcoholic Fatty Liver Disease or Fatty Liver, Nonalcoholic or Fatty Livers, Nonalcoholic or Liver, Nonalcoholic Fatty or Livers, Nonalcoholic Fatty or Nonalcoholic Fatty Liver or Nonalcoholic Fatty Livers or Nonalcoholic Steatohepatitis or Nonalcoholic Steatohepatitides or Steatohepatitides, Nonalcoholic or Steatohepatitis, Nonalcoholic).af. 96,426

3 (Metabolic Associated Fatty Liver Disease or MAFLD or Metabolic Dysfunction-associated Steatotic Liver Disease or MASLD or Metabolic Associated Steatohepatitis or MASH).af. 15,772

4 randomized.ab,ti. 1,291,151

5 randomised.ab,ti. 240,831

6 placebo.ab,ti. 462,253

7 randomly.ab,ti. 661,979

8 trial.ab,ti. 1,475,083

9 drug therapy.sh. 1,539,455

10 randomized controlled trial.pt,sh. 1,095,179

11 controlled clinical trial.pt,sh. 460,019

12 4 or 5 or 6 or 7 or 8 or 9 or 10 or 11 4,187,046

13 1 and (2 or 3) and 12 398

Cochrane Library: 118 results

Date of search: August 23, 2025

#1 (semaglutide):ti,ab,kw OR (rybelsus):ti,ab,kw OR (Ozempic):ti,ab,kw 1,641

#2 semaglutide 1,663

#3 #1 OR #2 1,670

#4 non-alcoholic steatohepatitis 1,487

#5 (Non-alcoholic Fatty Liver Disease):ti,ab,kw OR (NAFLD):ti,ab,kw OR (Nonalcoholic Fatty Liver Disease):ti,ab,kw OR (Fatty Liver, Nonalcoholic):ti,ab,kw OR (Fatty Livers, Nonalcoholic):ti,ab,kw 5,055

#6 (Liver, Nonalcoholic Fatty):ti,ab,kw OR (Livers, Nonalcoholic Fatty):ti,ab,kw OR (Nonalcoholic Fatty Liver):ti,ab,kw OR (Nonalcoholic Fatty Livers):ti,ab,kw OR (Nonalcoholic Steatohepatitis):ti,ab,kw 5,271

#7 (Nonalcoholic Steatohepatitides):ti,ab,kw OR (Steatohepatitides, Nonalcoholic):ti,ab,kw OR (Steatohepatitis, Nonalcoholic):ti,ab,kw 1,978

#8 #4 OR #5 OR #6 OR #7 5,593

#9 Metabolic Associated Fatty Liver Disease 1,004

#10 (MAFLD):ti,ab,kw (Metabolic Dysfunction-associated Steatotic Liver Disease):ti,ab,kw OR (MASLD):ti,ab,kw OR (Metabolic Associated Steatohepatitis):ti,ab,kw OR (MASH):ti,ab,kw 743

#11 #9 OR #10 1,311

#12 #8 OR #11 6,246

#13 #3 and #12 in Trials 118
