# Supplementary material for: The impact of semaglutide on liver outcomes in patients with or at risk of MASH: a dose and duration response meta-analysis of randomized trials
Source: Diabetol Metab Syndr. 2025 Nov 24;17:439. doi: 10.1186/s13098-025-01995-z (PMC12642090; doi:10.1186/s13098-025-01995-z)
Supplement: Supplementary file 2 — Supplementary Material 2 [file 13098_2025_1995_MOESM2_ESM.pdf]

Intention-to-treat

Study ID	Experimental	Comparator	Outcome	Weight	D1	D2	D3	D4	D5	Overall
Romero-Gomez et al, 2023	semaglutide	efinopegdutide	steatosis, liver biochemistry, metabolic outcomes	1	+	-	+	+	+	-
Davies et al, 2017	semaglutide	placebo	metabolic outcomes	1	+	!	+	+	+	!
Davies et al, 2021	semaglutide	placebo	liver biochemistry, metabolic outcomes	1	+	+	+	+	+	+
Nauck et al, 2016	semaglutide	placebo	liver biochemistry, metabolic outcomes	1	+	+	+	+	+	+
Alkhouri et al, 2022	semaglutide	combined therapy	steatosis, fibrosis, liver biochemistry	1	+	+	+	+	+	+
Newsome et al, 2021	semaglutide	placebo	NASH resolution, liver chemistry	1	+	+	+	+	+	+
O'Neil et al, 2018	semaglutide	placebo	metabolic outcomes	1	+	+	+	+	+	+
Pratley et al, 2019	Oral semaglutide	placebo	metabolic outcomes	1	+	+	+	+	+	+
Loomba et al, 2023	semaglutide	placebo	NASH resolution, liver biochemistry	1	+	+	+	+	+	+
Marso et al, 2016	semaglutide	placebo	metabolic outcomes	1	+	+	+	+	+	+
Wadden et al, 2021	semaglutide	placebo	metabolic outcomes	1	+	+	+	+	+	+
Kimura et al, 2023	semaglutide	dulaglutide	liver biochemistry, metabolic outcomes	1	+	!	+	+	+	!
Garvey et al, 2022	semaglutide	placebo	metabolic outcome	1	+	+	+	+	+	+
Seino et al, 2018	semaglutide	sitagliptin	metabolic outcomes	1	+	!	+	+	+	!
Lincoff et al, 2023	semaglutide	placebo	metabolic outcomes	1	+	+	+	+	+	+
Ahmann et al, 2018	semaglutide	exenatide	metabolic outcomes	1	+	!	+	+	+	!
Flint et al, 2021	semaglutide	placebo	steatosis, stiffness, liver biochemistry	1	+	+	!	+	+	!
Rubino et al, 2021	semaglutide	placebo	metabolic outcomes	1	+	+	+	+	+	+
Knop et al, 2023	oral semaglutide	placebo	metabolic outcomes	1	+	+	+	+	+	+
Wilding et al, 2021	semaglutide	placebo	metabolic outcomes	1	+	+	+	+	+	+
Frias et al, 2021	semaglutide	tirzepatide	liver biochemistry, metabolic outcomes	1	+	!	+	+	+	!
Sanyal et al, 2025	semaglutide	placebo	MASH resolution, liver biochemistry	1	+	+	+	+	+	+

+ Low risk

! Some concerns

- High risk

D1 Randomisation process

D2 Deviations from the intended interventions

D3 Missing outcome data

D4 Measurement of the outcome

D5 Selection of the reported result
