# Supplementary material for: The impact of semaglutide on liver outcomes in patients with or at risk of MASH: a dose and duration response meta-analysis of randomized trials
Source: Diabetol Metab Syndr. 2025 Nov 24;17:439. doi: 10.1186/s13098-025-01995-z (PMC12642090; doi:10.1186/s13098-025-01995-z)
Supplement: Supplementary file 3 — Supplementary Material 3 [file 13098_2025_1995_MOESM3_ESM.pdf]

Author(s): Ranran Kan
Question: Semaglutide compared to control for MASH
Setting:
Bibliography:

Certainty assessment							№ of patients		Effect		Certainty	Importance
№ of studies	Study design	Risk of bias	Inconsistency	Indirectness	Imprecision	Other considerations	semaglutide	control	Relative (95% CI)	Absolute (95% CI)		
**MASH resolution (follow-up: range 48 weeks to 72 weeks; assessed with: RR)**												
3	randomised trials	not serious	not serious	not serious	not serious	none	487/821 (59.3%)	113/370 (30.5%)	**RR 1.98** (1.57 to 2.50)	**299 more per 1,000** (from 174 more to 458 more)	⊕⊕⊕⊕ High	CRITICAL
								20.0%		**196 more per 1,000** (from 114 more to 300 more)		
**Improvement in liver fibrosis (follow-up: range 48 to 72; assessed with: RR)**												
3	randomised trials	not serious	serious[a]	not serious	not serious	none	326/821 (39.7%)	100/370 (27.0%)	**RR 1.18** (0.74 to 1.88)	**49 more per 1,000** (from 70 fewer to 238 more)	⊕⊕⊕◯ Moderate[a]	CRITICAL
								20.0%		**36 more per 1,000** (from 52 fewer to 176 more)		
**liver steatosis (follow-up: range 32 weeks to 72 weeks; assessed with: MD)**												
4	randomised trials	serious[b]	serious[a]	not serious	not serious	strong association	394	209	-	MD **11.3 lower** (18.7 lower to 3.91 lower)	⊕⊕⊕◯ Moderate[a,b]	IMPORTANT
**liver stiffness (follow-up: range 35 weeks to 72 weeks; assessed with: MD)**												
5	randomised trials	serious[c]	not serious	not serious	not serious	none	876	500	-	MD **0.88 lower** (1.91 lower to 0.15 higher)	⊕⊕⊕◯ Moderate[c]	IMPORTANT
**fibrosis test score (follow-up: range 35 weeks to 72 weeks)**												
3	randomised trials	serious[c]	serious[a]	not serious	not serious	none	295	200	-	MD **0.26 lower** (0.63 lower to 0.11 higher)	⊕⊕◯◯ Low[a,c]	IMPORTANT
**enhanced liver fibrosis (assessed with: MD)**												
4	randomised trials	serious[b]	not serious	not serious	not serious	none	868	505	-	MD **0.49 lower** (0.29 lower to 0.7 lower)	⊕⊕⊕◯ Moderate[b]	IMPORTANT
**ALT (follow-up: range 12 weeks to 72 weeks; assessed with: MD)**												
10	randomised trials	serious[b,c]	serious[a]	not serious	not serious	strong association dose response gradient	2513	1494	-	MD **5.55 - lower** (9.21 lower to 1.89 lower)	⊕⊕⊕⊕ High[a,b,c]	IMPORTANT

CI: confidence interval; **MD:** mean difference; **RR:** risk ratio

Explanations

a. Significant heterogeneity
b. The proportion of the number of people lost to follow-up in the two groups is mildly unbalanced in Romero-Gomez et al.
c. The proportion of people lost to follow-up is mildly unbalanced in Flint et al.
