# Supplementary material for: The impact of semaglutide on liver outcomes in patients with or at risk of MASH: a dose and duration response meta-analysis of randomized trials
Source: Diabetol Metab Syndr. 2025 Nov 24;17:439. doi: 10.1186/s13098-025-01995-z (PMC12642090; doi:10.1186/s13098-025-01995-z)
Supplement: Supplementary file 4 — Supplementary Material 4 [file 13098_2025_1995_MOESM4_ESM.docx]

**Supplementary Table S1.** Results of Univariable Meta-Regression Analyses of Potential Factors Influencing AST Level.

| Covariate | No. of Studies | Coefficient | | 95% CI | P-  value | Adjusted R^2^ (%) |
| --- | --- | --- | --- | --- | --- | --- |
| Dose of semaglutide, mg/w | 9 | -3.99 | (-8.13 to 0.15) | | 0.058 | 17.49 |
| Baseline BMI, kg/m^2^ | 9 | -1.64 | (-4.81 to 1.54) | | 0.262 | 3.28 |
| Baseline weight, kg | 9 | -0.39 | (-1.42 to 0.63) | | 0.396 | -5.89 |
| Mean age, y | 9 | -0.48 | (-2.39 to 1.43) | | 0.571 | -10.84 |
| Double-blind (Yes vs. No) | 9 | -7.34 | (-20.12 to 5.44) | | 0.217 | 6.10 |
| Active-control (Yes vs. No) | 9 | 7.34 | (-5.44 to 20.12) | | 0.217 | 6.10 |
| Baseline HbA_1c_, % | 9 | 4.92 | (-3.36 to 13.20) | | 0.203 | 10.45 |
| Baseline FPG, mmol/L | 9 | 0.36 | (-7.81 to 8.52) | | 0.921 | -21.60 |
| Duration of treatment, w | 9 | -0.14 | (-0.42 to 0.13) | | 0.263 | 3.62 |
| Sample size | 9 | <0.01 | (-0.01 to 0.01) | | 0.798 | -19.71 |

Abbreviation: AST, aspartate aminotransferase; CI, confidence interval; BMI, body mass index; HbA1c, glycated hemoglobin; FPG, fasting plasma glucose.

**P* < 0.05, ** *P* < 0.01, *** *P* < 0.001, **** *P* < 0.0001.


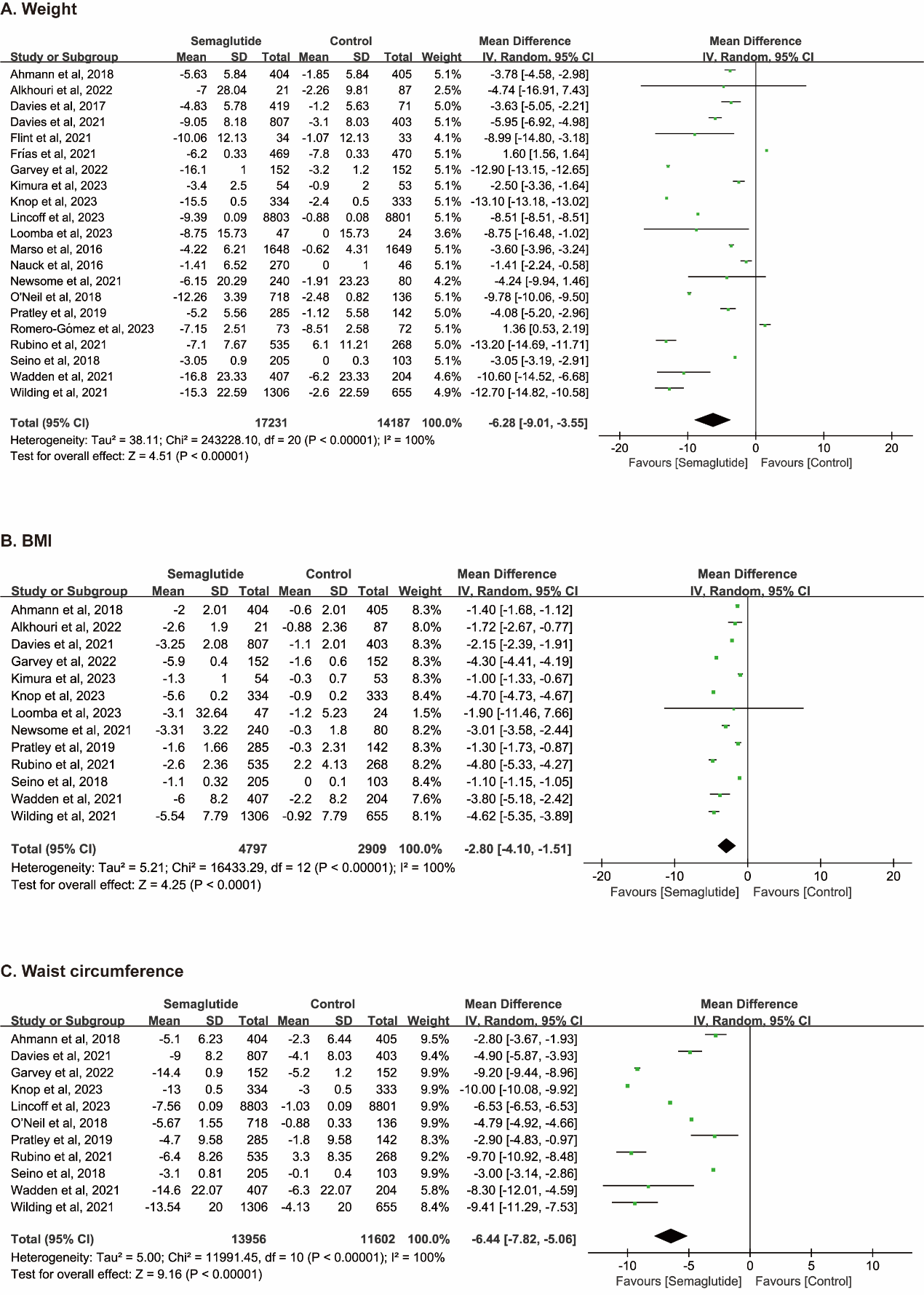


Figure S1. Meta-analyses of effect of Semaglutide on weight profile including (A) weight, (B) BMI, (C) waist circumference from baseline in adults with MASH.


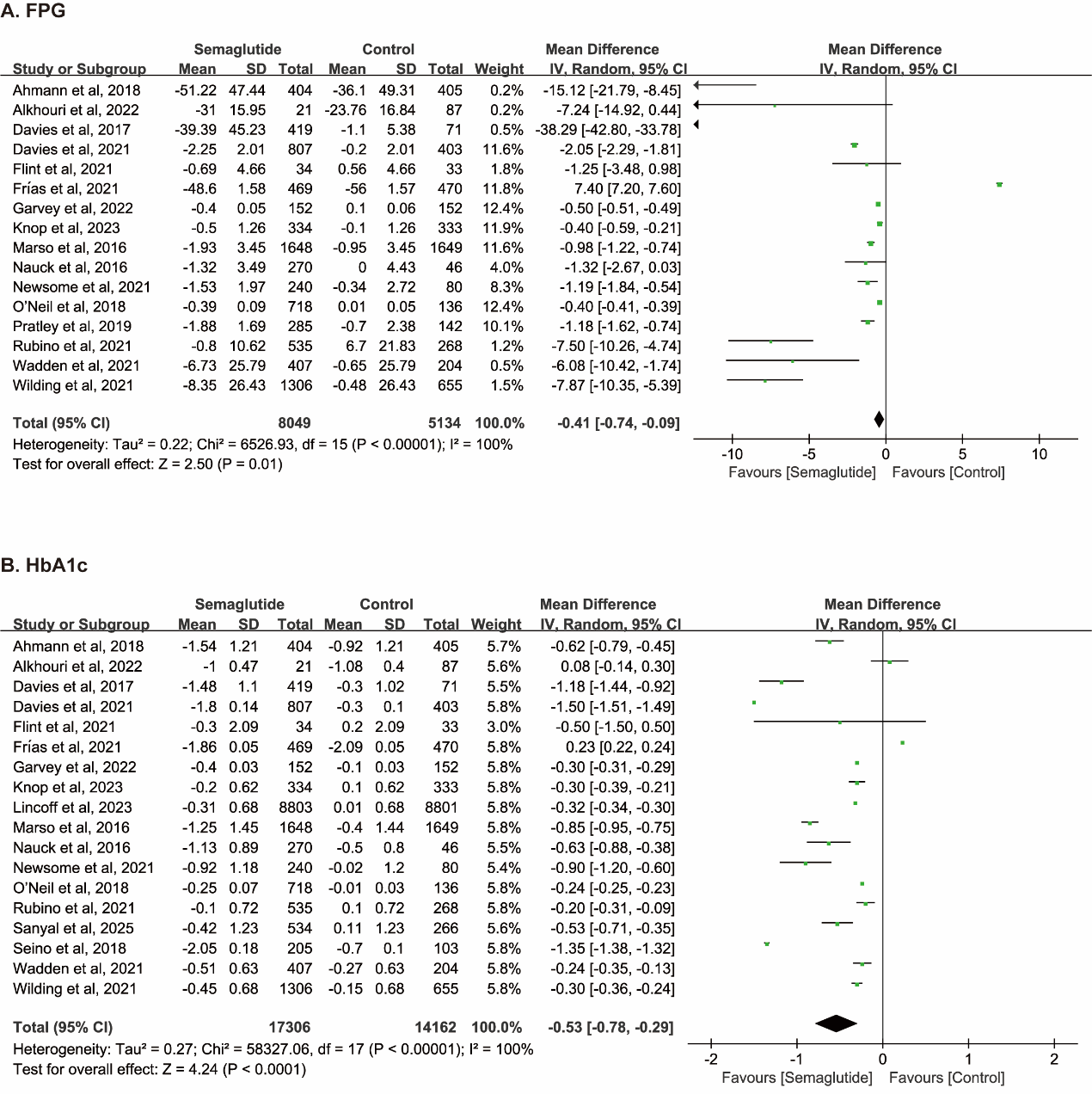


Figure S2. Meta-analyses of effect of Semaglutide on glycemic control including (A) fasting plasma glucose (FPG) and (B) glycosylated hemoglobin type A1c (HbA1c) from baseline in adults with MASH.


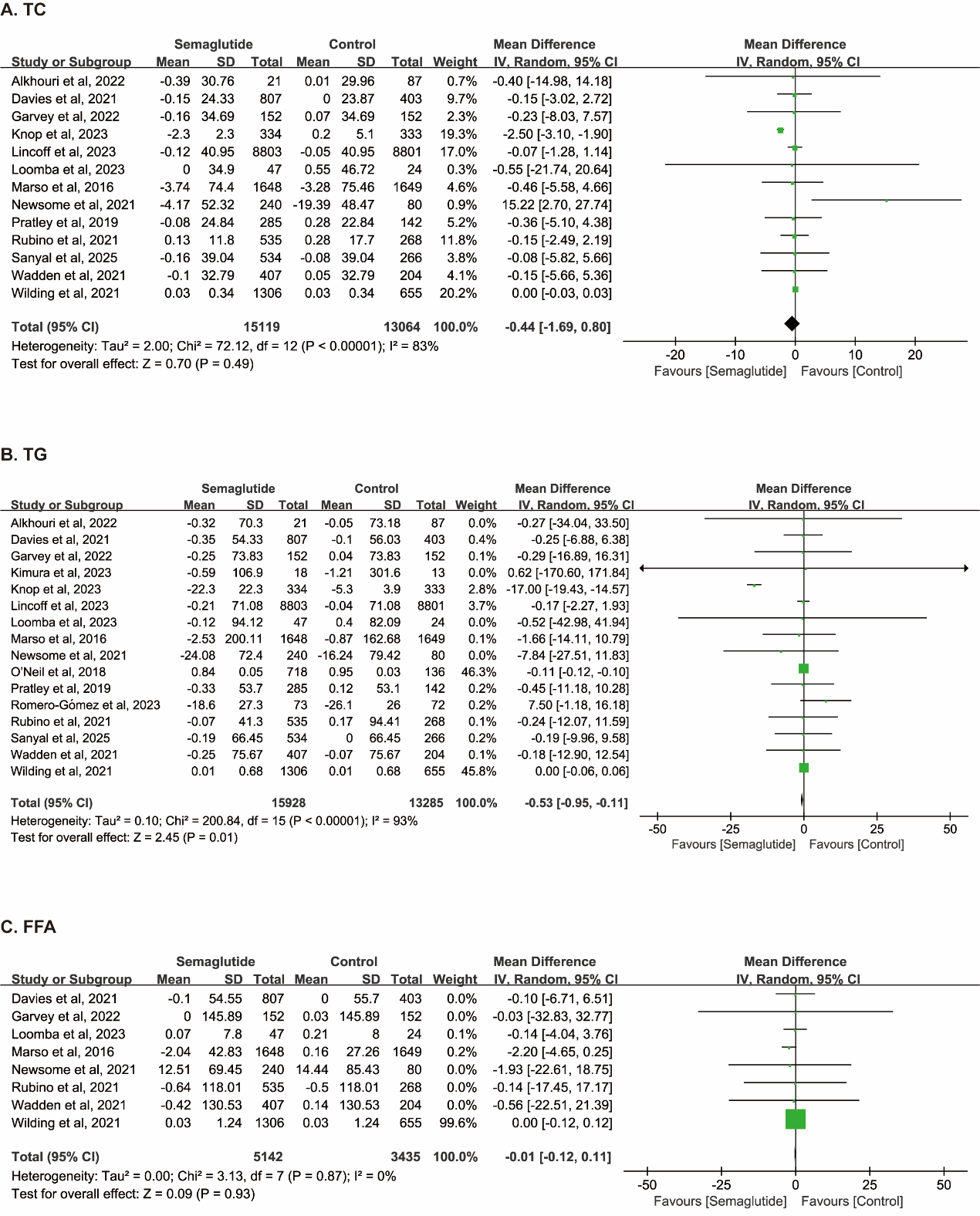


Figure S3. Meta-analyses of changes in concentration of (A) total cholesterol (TC) , (B) triglycerides (TG), and (C) free fatty acid (FFA) in adults with MASH compared Semaglutide and controls.


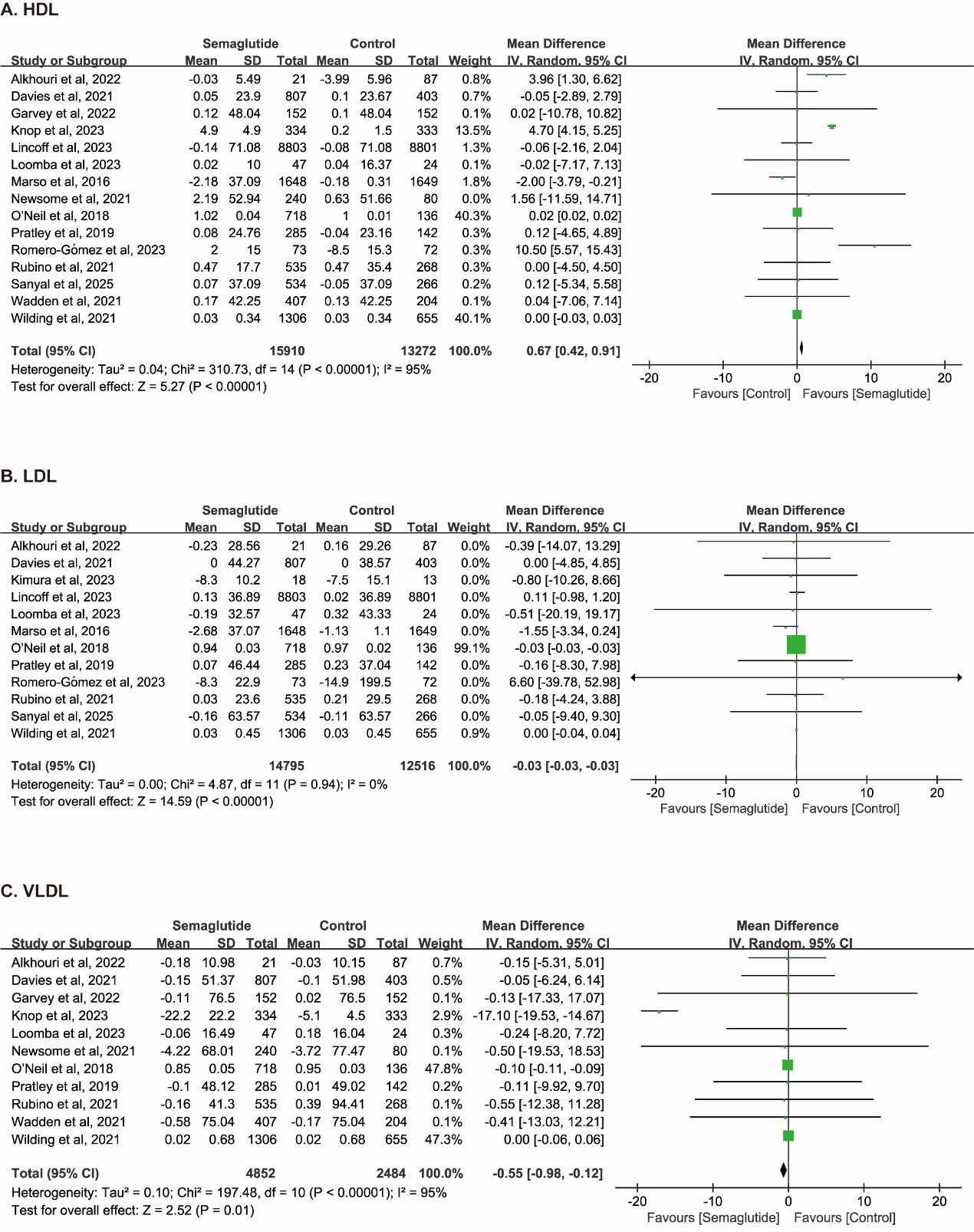


Figure S4. Meta-analyses of changes in concentration of (A) high-density lipoprotein cholesterol (HDL), (B) low-density lipoprotein cholesterol (LDL), and (C) very low-density lipoprotein cholesterol (VLDL) from baseline in adults with MASH compared Semaglutide and controls.


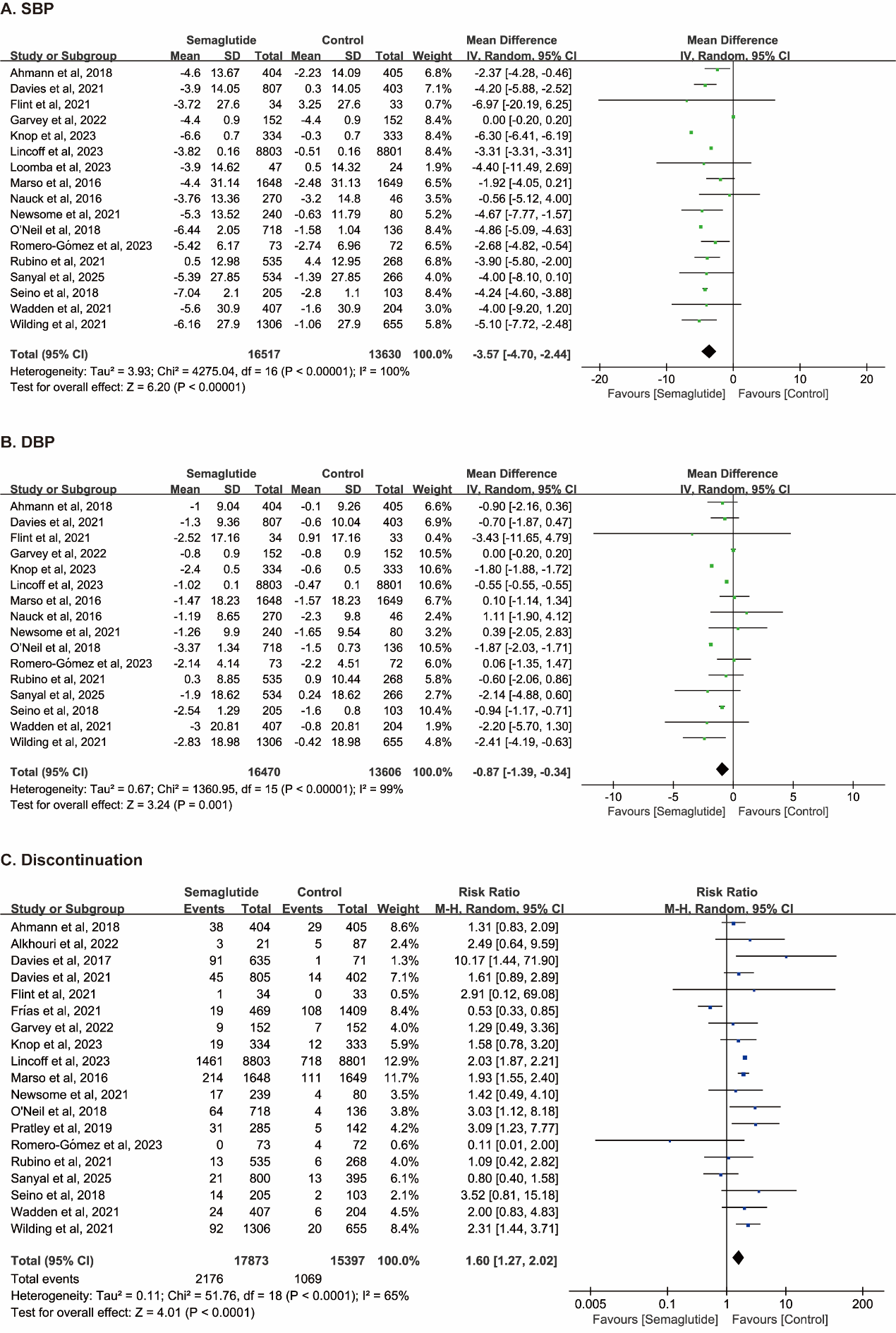


Figure S5. Meta-analyses of effect of Semaglutide on (A) systolic blood pressure (SBP) and (B) diastolic blood pressure (DBP) from baseline in adults with MASH, and the incidences of (C) trial discontinuation in adults with MASH comparing Semaglutide with controls.


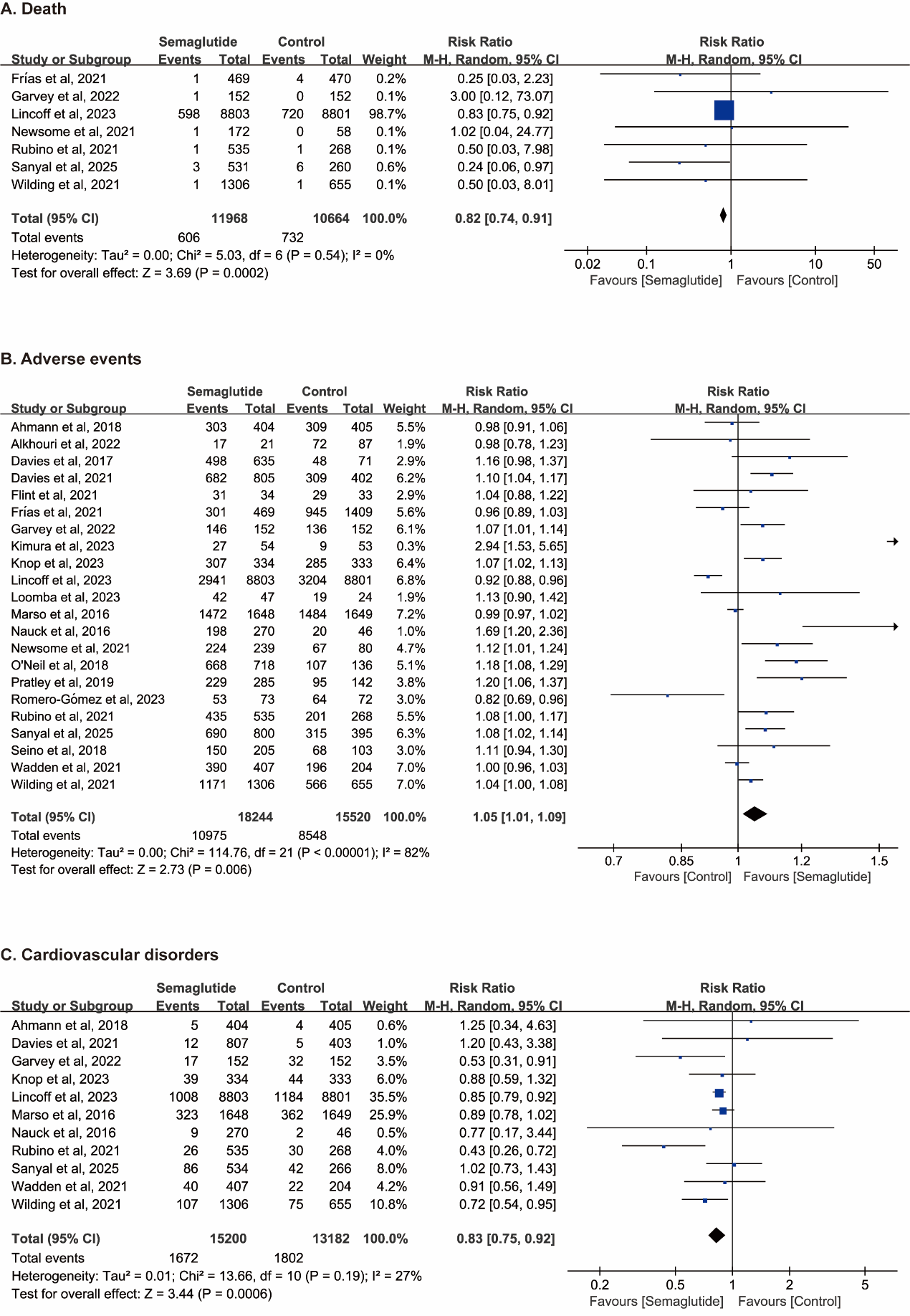


Figure S6. Meta-analyses of incidences of (A) death, (B) adverse events, and (C) cardiovascular disorders in adults with MASH comparing Semaglutide with controls.


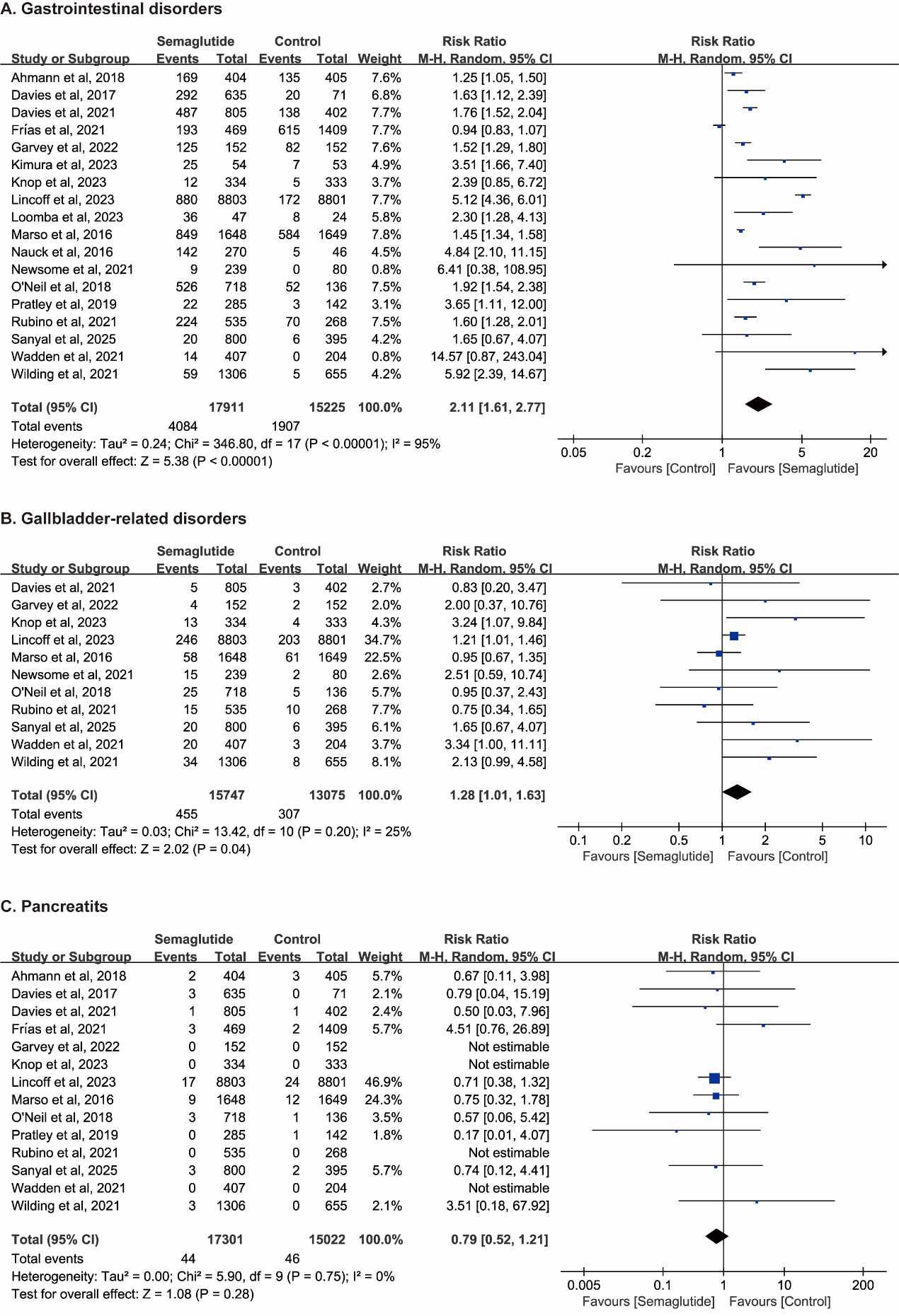


Figure S7. Meta-analyses of incidences of (A) gastrointestinal disorders, (B) gallbladder-related disorders, and (C) pancreatitis in adults with MASH comparing Semaglutide with controls.


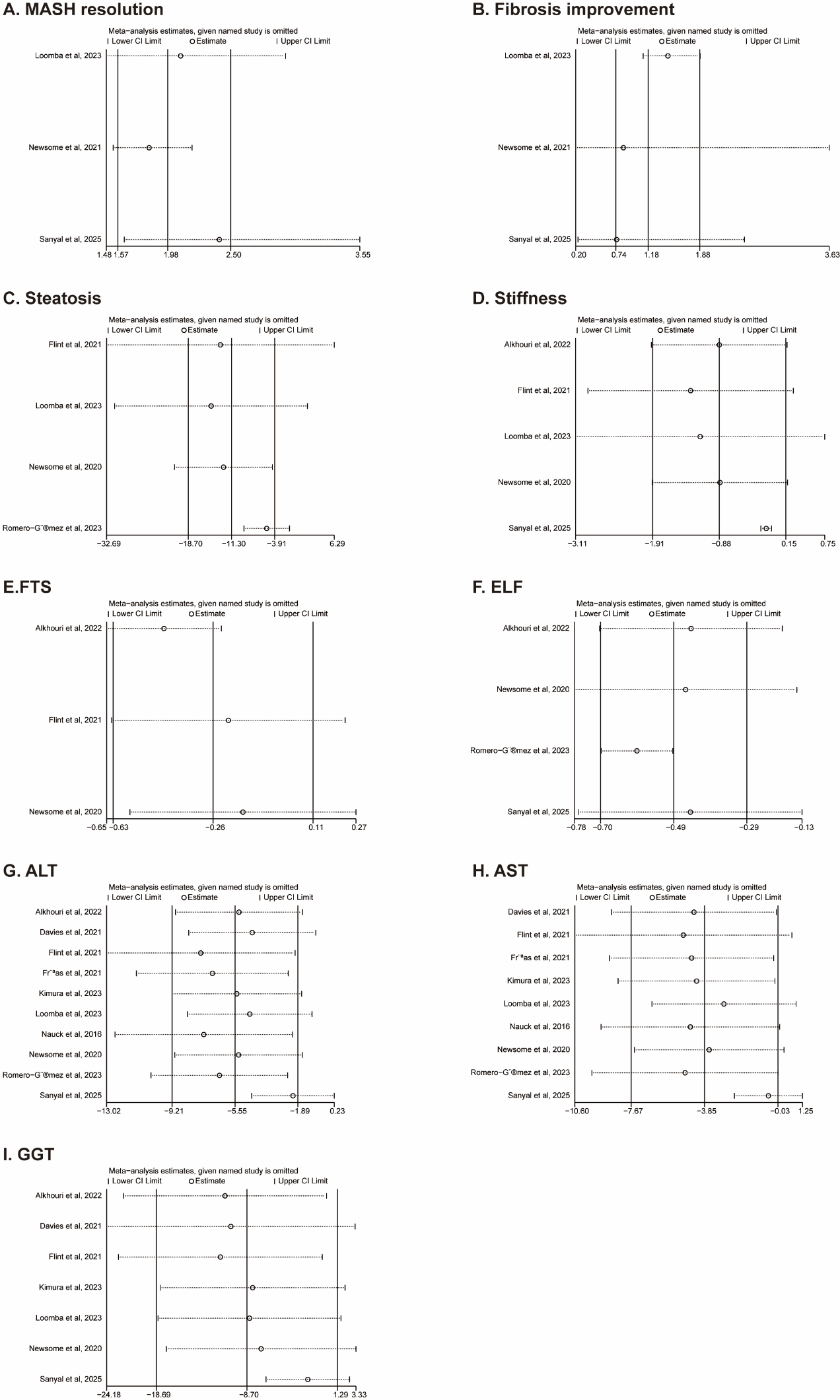


Figure S8. Sensitivity analyses of effect of Semaglutide on (A) resolution of MASH, (B) improvement in liver fibrosis, (C) steatosis, (D) stiffness, (E) fibrosis test score(FTS) and (F) enhanced liver fibrosis(ELF), (G) ALT, (H) AST and (I) GGT in adults with MASH.


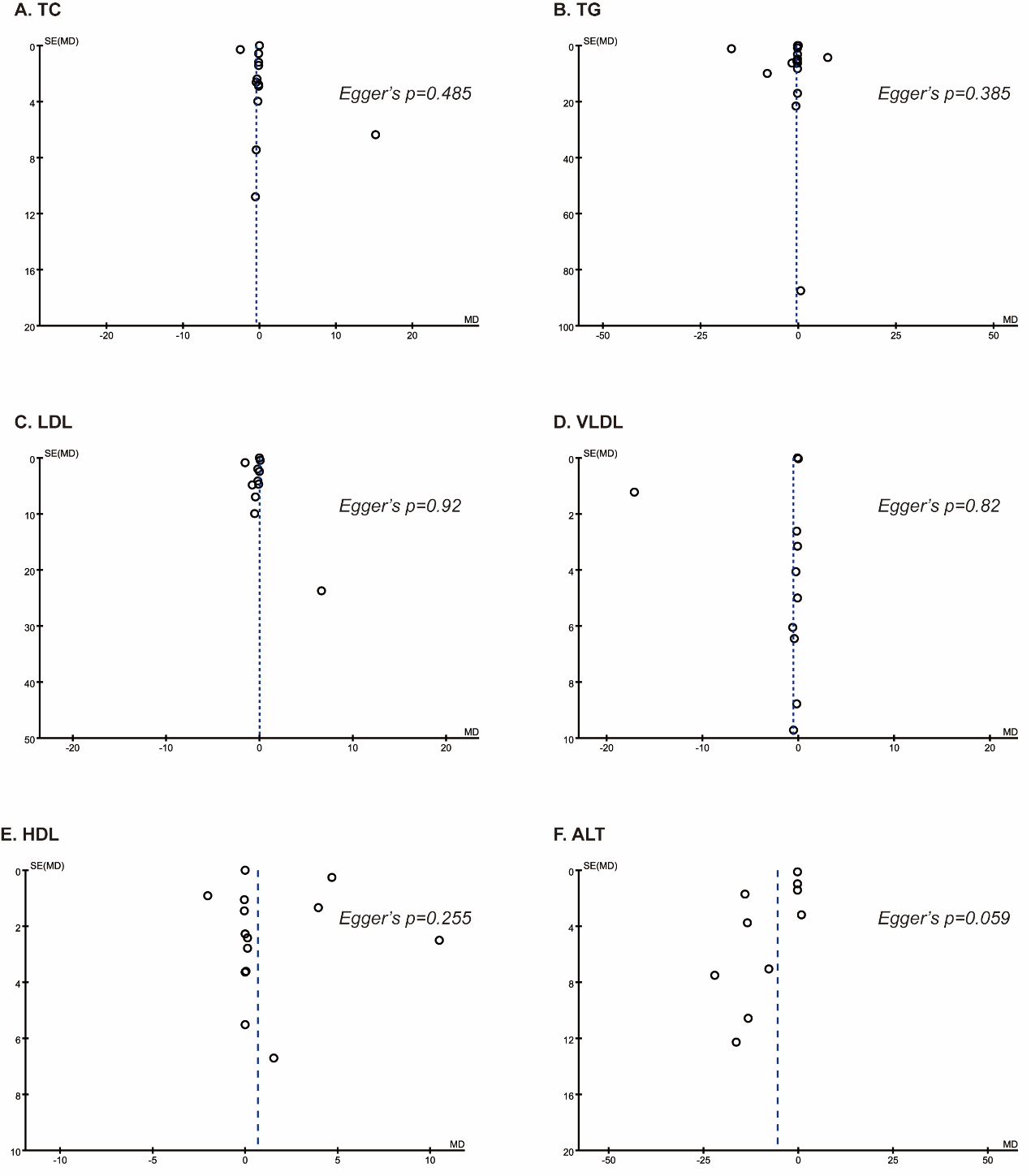


Figure S9. Egger’s test for publication bias on (A) TC, (B) TG, (C) LDL, (D) VLDL, (E) HDL and (F) ALT in adults with MASH.


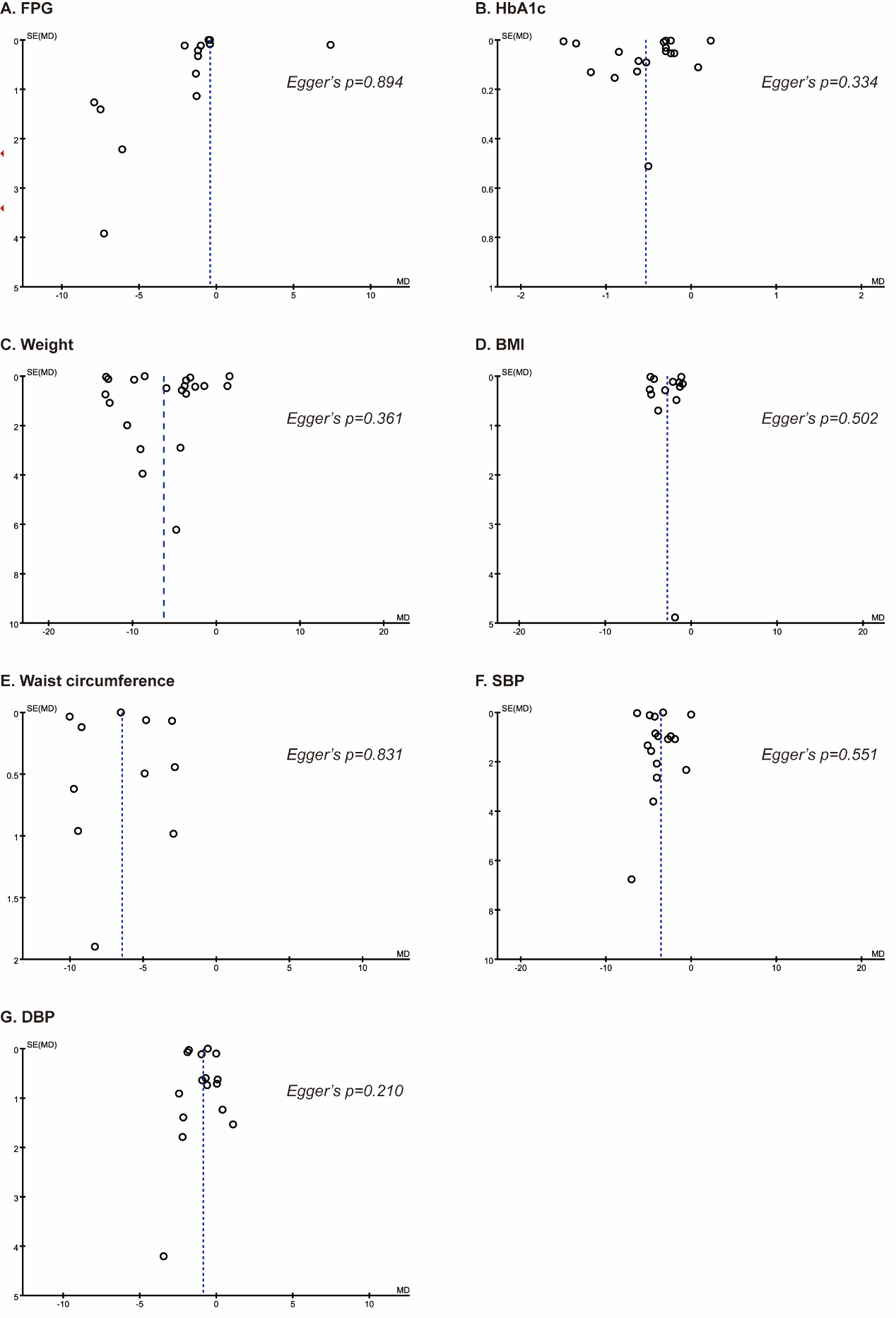


Figure S10. Egger’s test for publication bias on (A) FPG, (B) HbA1c, (C) weight, (D) BMI, (E) waist circumference, (F) SBP and (G) DBP in adults with MASH.


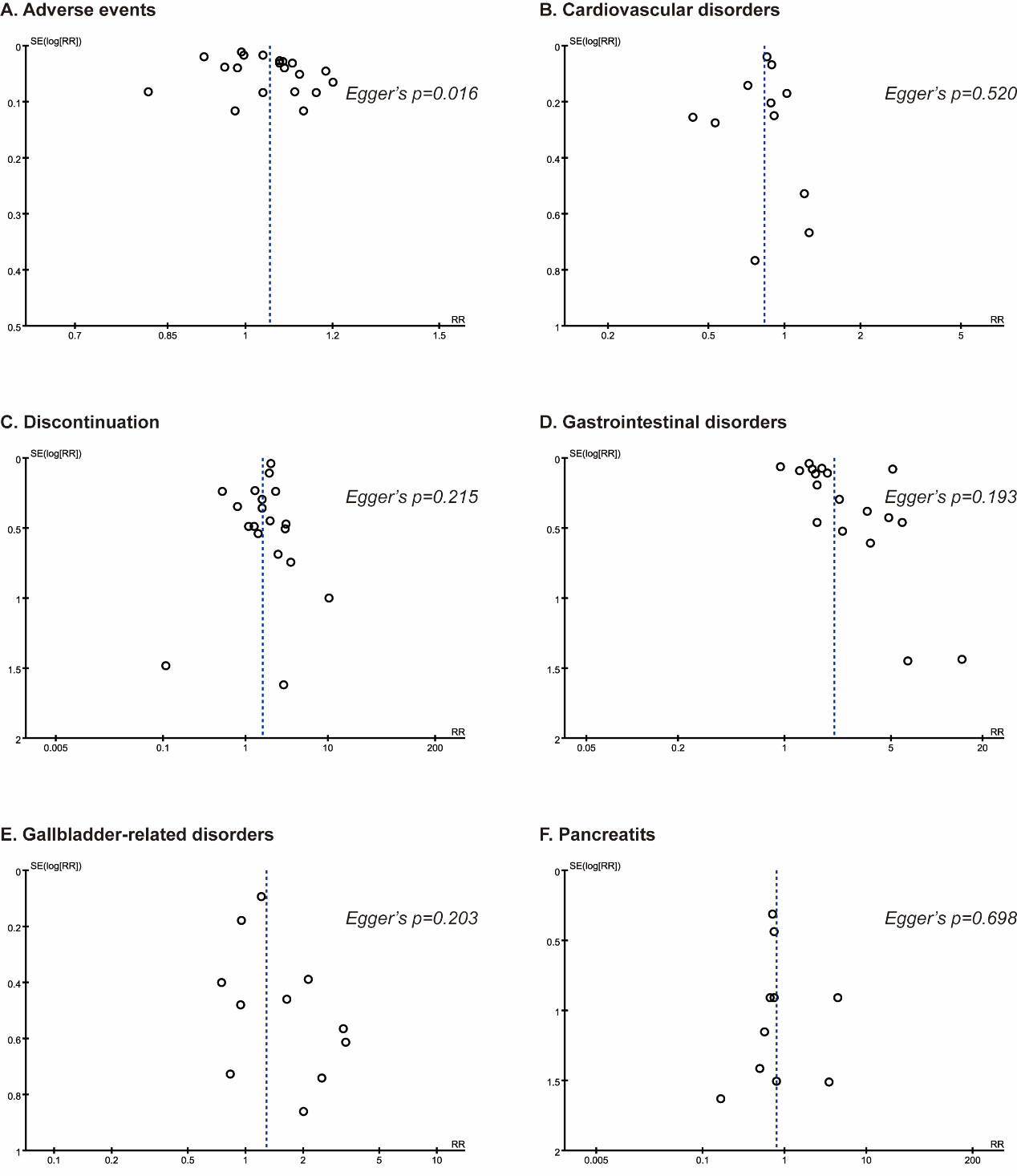


Figure S11. Egger’s test for publication bias on (A) adverse events, (B) cardiovascular disorders, (C) discontinuation, (D) gastrointestinal disorders, (E)gallbladder-related disorders and (F) pancreatitis in adults with MASH.
