# Supplementary material for: The impact of semaglutide on liver outcomes in patients with or at risk of MASH: a dose and duration response meta-analysis of randomized trials
Source: Diabetol Metab Syndr. 2025 Nov 24;17:439. doi: 10.1186/s13098-025-01995-z (PMC12642090; doi:10.1186/s13098-025-01995-z)
Supplement: Supplementary file 5 — Supplementary Material 5 [file 13098_2025_1995_MOESM5_ESM.docx]

**Supplementary Table S1.**

Comparison of Original and Revised Pooled Estimates for Primary Outcomes.

| Outcome | Original Effect Measure | Original Model | Effect Size (95% CI) | P-value | Revised Effect Measure | Revised Model | Effect Size (95% CI) | P-value |
| --- | --- | --- | --- | --- | --- | --- | --- | --- |
| Steatosis (dB) | SMD | Random-effects | -1.05 (-1.94 to -0.16) | 0.02 | MD | Random-effects | -11.30 (-18.70 to -3.91) | 0.003 |
| Liver stiffness (kPa) | SMD | Fixed-effects | -0.27 (-0.59 to 0.05) | 0.10 | MD | Random-effects | -0.88 (-1.91 to 0.15) | 0.09 |
| FTS* | SMD | Random-effects | -0.37 (-0.57 to -0.16) | 0.0004 | MD | Random-effects | -0.26 (-0.63 to 0.11) | 0.17 |
| ELF | SMD | Random-effects | -0.60 (-1.08 to -0.11) | 0.02 | MD | Random-effects | -0.49 (-0.70 to -0.29) | <0.0001 |
| ALT (U/L) | SMD | Fixed-effects | -0.13 (-0.21 to -0.06) | 0.0005 | MD | Random-effects | -5.55 (-9.21 to -1.89) | 0.003 |
| AST (U/L) | SMD | Fixed-effects | -0.04 (-0.11 to 0.04) | 0.31 | MD | Random-effects | -3.85 (-7.67 to -0.03) | 0.05 |
| GGT (U/L)* | SMD | Fixed-effects | -0.12 (-0.22 to -0.02) | 0.02 | MD | Random-effects | -8.7 (-18.69 to 1.29) | 0.09 |

Abbreviations: FTS, fibrosis test score; ELF, enhanced liver fibrosis score; ALT, alanine aminotransferase; AST, aspartate aminotransferase; GGT, gamma-glutamyl transferase; MD, mean difference; SMD, standardized mean difference; CI, confidence interval.

*Indicates outcomes for which the statistical significance (P-value threshold of 0.05) changed between the original and revised analyses.

The revised analyses incorporate one additional eligible study (Sanyal et al., 2025).

Negative effect sizes indicate a decrease favoring the semaglutide group.

**Supplementary Table S2.**

Results of univariable meta-regression analyses of potential factors influencing AST level.

| Covariate | No. of Studies | Coefficient | | 95% CI | P-value | Adjusted R^2^ (%) |
| --- | --- | --- | --- | --- | --- | --- |
| Dose of semaglutide, mg/w | 9 | -3.99 | (-8.13 to 0.15) | | 0.058 | 17.49 |
| Baseline BMI, kg/m^2^ | 9 | -1.64 | (-4.81 to 1.54) | | 0.262 | 3.28 |
| Baseline weight, kg | 9 | -0.39 | (-1.42 to 0.63) | | 0.396 | -5.89 |
| Mean age, y | 9 | -0.48 | (-2.39 to 1.43) | | 0.571 | -10.84 |
| Double-blind (Yes vs. No) | 9 | -7.34 | (-20.12 to 5.44) | | 0.217 | 6.10 |
| Active-control (Yes vs. No) | 9 | 7.34 | (-5.44 to 20.12) | | 0.217 | 6.10 |
| Baseline HbA_1c_, % | 9 | 4.92 | (-3.36 to 13.20) | | 0.203 | 10.45 |
| Baseline FPG, mmol/L | 9 | 0.36 | (-7.81 to 8.52) | | 0.921 | -21.60 |
| Duration of treatment, w | 9 | -0.14 | (-0.42 to 0.13) | | 0.263 | 3.62 |
| Sample size | 9 | <0.01 | (-0.01 to 0.01) | | 0.798 | -19.71 |

Abbreviation: AST, aspartate aminotransferase; CI, confidence interval; BMI, body mass index; HbA1c, glycated hemoglobin; FPG, fasting plasma glucose.

**P* < 0.05, ** *P* < 0.01, *** *P* < 0.001, **** *P* < 0.0001.

**Supplementary Table S3.**

Subgroup Analyses of Safety Outcomes by Weekly Dose of Subcutaneous Semaglutide.

| Subgroups | No. of Studies | Risk Ratio  (95% CI) | *I^2^* | Test for  Subgroup Differences |
| --- | --- | --- | --- | --- |
| **Discontinuation** | **16** | **1.58 (1.24 to 2.02)** | **68%** |  |
| < 1.0 mg | 2 | 1.97 (1.59 to 2.44) | 0% | Chi^2^ = 1.07, df = 2 (P = 0.58) |
| 1.0-1.9 mg | 6 | 1.45 (0.78 to 2.69) | 74% |  |
| ≥ 2.0 mg | 8 | 1.76 (1.35 to 2.29) | 30% |  |
| **Mortality** | **7** | **0.82 (0.74 to 0.91)** | **0%** |  |
| 1.0-1.9 mg | 2 | 0.39 (0.06 to 2.39) | 0% | Chi^2^ = 0.65, df = 1 (P = 0.42) |
| ≥ 2.0 mg | 5 | 0.82 (0.74 to 0.91) | 0% |  |
| **Adverse Events** | **20** | **1.04 (1.01 to 1.08)** | **82%** |  |
| < 1.0 mg | 3 | 1.18 (0.96 to 1.44) | 92% | Chi^2^ = 1.57, df = 2 (P = 0.46) |
| 1.0-1.9 mg | 8 | 1.05 (0.96 to 1.14) | 78% |  |
| ≥ 2.0 mg | 9 | 1.03 (0.97 to 1.09) | 87% |  |
| **Cardiovascular Events** | **10** | **0.82 (0.73 to 0.93)** | **34%** |  |
| < 1.0 mg | 2 | 0.89 (0.78 to 1.02) | 0% | Chi^2^ = 2.46, df = 2 (P = 0.29) |
| 1.0-1.9 mg | 2 | 1.22 (0.54 to 2.75) | 0% |  |
| ≥ 2.0 mg | 6 | 0.76 (0.62 to 0.93) | 58% |  |
| **Gastrointestinal Disorders** | **16** | **2.06 (1.56 to 2.73)** | **96%** |  |
| < 1.0 mg | 3 | 1.92 (1.33 to 2.76) | 85% | Chi^2^ = 2.62, df = 2 (P = 0.27) |
| 1.0-1.9 mg | 6 | 1.52 (1.09 to 2.13) | 90% |  |
| ≥ 2.0 mg | 7 | 2.70 (1.42 to 5.12) | 96% |  |
| **Gallbladder-Related Disorders** | **10** | **1.21 (0.98 to 1.48)** | **13%** |  |
| < 1.0 mg | 2 | 0.95 (0.68 to 1.32) | 0% | Chi^2^ = 2.66, df = 2 (P = 0.26) |
| 1.0-1.9 mg | 2 | 1.43 (0.48 to 4.27) | 13% |  |
| ≥ 2.0 mg | 6 | 1.39 (1.00 to 1.93) | 27% |  |
| **Pancreatitis** | **12** | **0.81 (0.53 to 1.25)** | **0%** |  |
| < 1.0 mg | 2 | 0.72 (0.32 to 1.62) | 0% | Chi^2^ = 0.88, df = 2 (P = 0.65) |
| 1.0-1.9 mg | 4 | 1.30 (0.45 to 3.79) | 0% |  |
| ≥ 2.0 mg | 6 | 0.76 (0.43 to 1.34) | 0% |  |

*Abbreviations:* CI, confidence interval.

Analyses are restricted to trials using subcutaneous administration of semaglutide; studies with oral administration were excluded due to difficulties in standardizing weekly dose equivalents.

Subgroups were defined as low dose (<1.0 mg/week), medium dose (1.0-1.9 mg/week), and high dose ≥ 2.0 mg/week). Heterogeneity was assessed using the *I^2^* statistic, and subgroup differences were tested using chi-square tests.

**Supplementary Table S4.**

Subgroup Analyses of Safety Outcomes by Intervention Duration.

| Subgroups | No. of Studies | Risk Ratio  (95% CI) | *I^2^* | Test for  Subgroup Differences |
| --- | --- | --- | --- | --- |
| **Discontinuation** | **19** | **1.60 (1.27 to 2.02)** | **65%** |  |
| Short-term (< 12 m) | 5 | 1.54 (0.41 to 5.81) | 79% | Chi^2^ = 0.07, df = 1 (P = 0.79) |
| Long-term (≥ 12 m) | 14 | 1.84 (1.61 to 2.11) | 18% |  |
| **Mortality** | **7** | **0.82 (0.74 to 0.91)** | **0%** |  |
| Short-term (< 12 m) | 1* | 0.25 (0.03 to 2.23) | None | Chi^2^ = 1.14, df = 1 (P = 0.29) |
| Long-term (≥ 12 m) | 6 | 0.82 (0.74 to 0.91) | 0% |  |
| **Adverse Events** | **22** | **1.05 (1.01 to 1.09)** | **82%** |  |
| Short-term (< 12 m) | 7 | 1.11 (0.94 to 1.31) | 82% | Chi^2^ = 0.39, df = 1 (P = 0.53) |
| Long-term (≥ 12 m) | 15 | 1.05 (1.01 to 1.09) | 84% |  |
| **Cardiovascular Events** | **11** | **0.83 (0.75 to 0.92)** | **27%** |  |
| Short-term (< 12 m) | 1* | 0.77 (0.17 to 3.44) | None | Chi^2^ = 0.07, df = 1 (P = 0.79) |
| Long-term (≥ 12 m) | 10 | 0.83 (0.74 to 0.93) | 34% |  |
| **Gastrointestinal Disorders** | **18** | **2.11 (1.61 to 2.77)** | **95%** |  |
| Short-term (< 12 m) | 4 | 2.07 (1.02 to 4.18) | 91% | Chi^2^ = 0.01, df = 1 (P = 0.92) |
| Long-term (≥ 12 m) | 14 | 2.15 (1.59 to 2.91) | 95% |  |
| **Pancreatitis** | **14** | **0.79 (0.52 to 1.21)** | **0%** |  |
| Short-term (< 12 m) | 2 | 2.83 (0.61 to 13.04) | 0% | Chi^2^ = 2.89, df = 1 (P = 0.09) |
| Long-term (≥ 12 m) | 12 | 0.71 (0.46 to 1.11) | 0% |  |

*Abbreviations:* CI, confidence interval.

Subgroups were defined as short-term (<12 months) and long-term (≥12 months) intervention.

Gallbladder-related disorders were not included in the duration subgroup analysis because all available data were from long-term studies (≥12 months).

Heterogeneity was assessed using the *I^2^* statistic, and subgroup differences were tested using chi-square tests.

*Indicates analyses with only one study, where heterogeneity assessment was not applicable.

**Supplementary Table S5.**

Sensitivity Analyses of Liver Outcomes Restricted to Trials with Histologically Confirmed MASH Populations.

| Outcome | Analysis | No. of Trials | No. of Participants | Effect Estimate  (95% CI) | *I^2^* | P value for  Heterogeneity |
| --- | --- | --- | --- | --- | --- | --- |
| MASH Resolution | Main analysis | 3 | 1,191 | 1.98 (1.57 to 2.50) | 20% | 0.29 |
|  | Sensitivity analysis | 3 | 1,191 | 1.98 (1.57 to 2.50) | 20% | 0.29 |
| Fibrosis Improvement | Main analysis | 3 | 1,191 | 1.18 (0.74 to 1.88) | 77% | 0.01 |
|  | Sensitivity analysis | 3 | 1,191 | 1.18 (0.74 to 1.88) | 77% | 0.01 |
| Liver Steatosis, % | Main analysis | 4 | 603 | -11.83 (-18.70 to -3.91) | 94% | < 0.00001 |
|  | Sensitivity analysis | 2 | 391 | -3.02 (-5.39 to -0.65) | 0% | 0.72 |
| Liver Stiffness, kPa | Main analysis | 5 | 1,376 | -0.88 (-1.91 to 0.15) | 85% | < 0.0001 |
|  | Sensitivity analysis | 4 | 1,299 | -1.33 (-2.92 to 0.27) | 75% | 0.007 |
| FTS | Main analysis | 3 | 495 | -0.26 (-0.63 to 0.11) | 88% | 0.0003 |
|  | Sensitivity analysis | 2 | 428 | -0.20 (-0.63 to 0.23) | 93% | 0.0002 |
| ELF | Main analysis | 4 | 1,373 | -0.49 (-0.70 to -0.29) | 76% | 0.006 |
|  | Sensitivity analysis | 3 | 1,228 | -0.60 (-0.70 to -0.50) | 0% | 0.87 |
| ALT, U/L | Main analysis | 10 | 4,007 | -5.55 (-9.21 to -1.89) | 90% | < 0.00001 |
|  | Sensitivity analysis | 4 | 1,299 | -14.50 (-17.68 to -11.31) | 0% | 0.77 |
| AST, U/L | Main analysis | 9 | 3,899 | -3.85 (-7.67 to -0.03) | 87% | < 0.00001 |
|  | Sensitivity analysis | 3 | 1,191 | -17.86 (-22.33 to -13.39) | 0% | 0.77 |
| GGT, U/L | Main analysis | 7 | 2,607 | -8.70 (-18.69 to 1.29) | 85% | < 0.00001 |
|  | Sensitivity analysis | 4 | 1,299 | -13.77 (-29.28 to 1.74) | 57% | 0.07 |

*Abbreviations:* MASH, metabolic dysfunction–associated steatohepatitis; FTS, Fibrosis Test score; ELF, Enhanced Liver Fibrosis score; ALT, alanine aminotransferase; AST, aspartate aminotransferase; GGT, gamma-glutamyl transferase.

Main analysis includes all eligible randomized controlled trials. Sensitivity analysis was restricted to studies enrolling participants with histologically confirmed MASH.

Effect estimates are presented as risk ratio (RR) for dichotomous outcomes and weighted mean difference (WMD) for continuous outcomes, with corresponding 95% confidence intervals (CI). Heterogeneity was assessed using the *I^2^* statistic and Cochran’s Q test.
